# How Modelers Model: the Overlooked Social and Human Dimensions in Model Intercomparison Studies

**DOI:** 10.1021/acs.est.2c02023

**Published:** 2022-09-02

**Authors:** Fabrizio Albanito, David McBey, Matthew Harrison, Pete Smith, Fiona Ehrhardt, Arti Bhatia, Gianni Bellocchi, Lorenzo Brilli, Marco Carozzi, Karen Christie, Jordi Doltra, Christopher Dorich, Luca Doro, Peter Grace, Brian Grant, Joël Léonard, Mark Liebig, Cameron Ludemann, Raphael Martin, Elizabeth Meier, Rachelle Meyer, Massimiliano De Antoni Migliorati, Vasileios Myrgiotis, Sylvie Recous, Renáta Sándor, Val Snow, Jean-François Soussana, Ward N. Smith, Nuala Fitton

**Affiliations:** † Institute of Biological and Environmental Sciences, School of Biological Science, University of Aberdeen, 23 Street Machar Drive, Aberdeen AB24 3UU, U.K.; ‡ Tasmanian Institute of Agriculture, University of Tasmania, Newnham Drive, Launceston, Tasmania 7248, Australia; § INRAE, CODIR, Paris 75007, France; ∥ RITTMO AgroEnvironnement, Colmar 68000, France; ⊥ ICAR-Indian Agricultural Research Institute, New Delhi 110012, India; # Université Clermont Auvergne, INRAE, VetAgro Sup, UREP, Clermont-Ferrand 63000, France; ¶ CNR-IBE, National Research Council Institute for the BioEconomy, Via Caproni 8, Florence 50145, Italy; ∇ UMR ECOSYS, INRAE, AgroParisTech, Université Paris-Saclay, Thiverval-Grignon 78850, France; ○ Tasmanian Institute of Agriculture, University of Tasmania, 16-20 Mooreville Road, Burnie, Tasmania 7320, Australia; ⧫ Sustainable Field Crops Programme, Institute of Agrifood Research and Technology (IRTA) Mas Badia, La Tallada d’Empordà, Girona 17134, Spain; †† Natural Resource Ecology Lab, Colorado State University, Fort Collins, Colorado 80521, United States; ‡‡ Texas A&M AgriLife Research, Blackland Research and Extension Center, Temple, Texas 76502, United States; §§ Desertification Research Centre, University of Sassari, Sassari 07100, Italy; ∥∥ Queensland University of Technology, Brisbane, Queensland 4000, Australia; ⊥⊥ Ottawa Research and Development Centre, Agriculture and Agri-Food Canada, Ottawa, Ontario K1A 0C6, Canada; ## BioEcoAgro Joint Research Unit, INRAE, Barenton-Bugny 02000, France; ¶¶ USDA-ARS Northern Great Plains Research Laboratory, P.O. Box 459, Mandan, North Dakota 58554, United States; ∇∇ Cameron Ludemann Consulting, Arnhem 6821 EV, The Netherlands; ○○ CSIRO Agriculture and Food, St Lucia, Queensland 4067, Australia; ⧫⧫ Faculty of Veterinary & Agricultural Sciences, University of Melbourne, Parkville, Victoria 3010, Australia; ††† Department of Environment and Science, Dutton Park, Queensland 4102, Australia; ‡‡‡ School of GeoSciences, University of Edinburgh, Edinburgh EH9 3JN, U.K.; §§§ Université de Reims Champagne-Ardenne, INRAE, FARE Laboratory, Reims 51100, France; ∥∥∥ Agricultural Institute, Centre for Agricultural Research, ELKH, Martonvásár 2462, Hungary; ⊥⊥⊥ AgResearch, P.O. Box 4749, Christchurch 8140, New Zealand

**Keywords:** model ensembles, biogeochemical models, multi-criteria decision-making, model calibration, model intercomparison, climate change, greenhouse gases, soil carbon, AgMIP

## Abstract

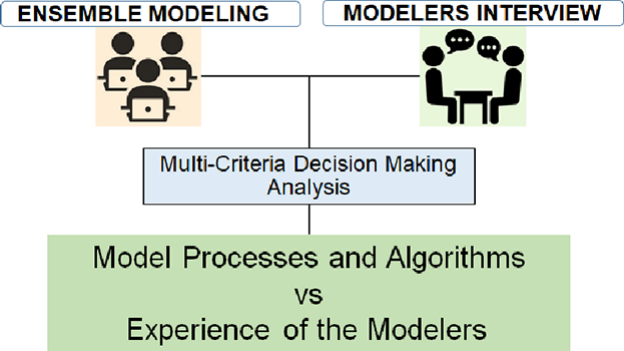

There is a growing realization that the complexity of model ensemble studies depends not only on the models used but also on the experience and approach used by modelers to calibrate and validate results, which remain a source of uncertainty. Here, we applied a multi-criteria decision-making method to investigate the rationale applied by modelers in a model ensemble study where 12 process-based different biogeochemical model types were compared across five successive calibration stages. The modelers shared a common level of agreement about the importance of the variables used to initialize their models for calibration. However, we found inconsistency among modelers when judging the importance of input variables across different calibration stages. The level of subjective weighting attributed by modelers to calibration data decreased sequentially as the extent and number of variables provided increased. In this context, the perceived importance attributed to variables such as the fertilization rate, irrigation regime, soil texture, pH, and initial levels of soil organic carbon and nitrogen stocks was statistically different when classified according to model types. The importance attributed to input variables such as experimental duration, gross primary production, and net ecosystem exchange varied significantly according to the length of the modeler’s experience. We argue that the gradual access to input data across the five calibration stages negatively influenced the consistency of the interpretations made by the modelers, with cognitive bias in “trial-and-error” calibration routines. Our study highlights that overlooking human and social attributes is critical in the outcomes of modeling and model intercomparison studies. While complexity of the processes captured in the model algorithms and parameterization is important, we contend that (1) the modeler’s assumptions on the extent to which parameters should be altered and (2) modeler perceptions of the importance of model parameters are just as critical in obtaining a quality model calibration as numerical or analytical details.

## Abstract 2

This study outlines subjective inconsistencies in the prioritization of variables used in model calibration, with implications in the outcomes of modeling and model intercomparison studies.

## Introduction

Multi-model ensemble comparisons are becoming increasingly common in contemporary research using agricultural simulation models to understand the impacts of weather variability,^1^ climate change,^2^ greenhouse gas (GHG) emissions from agriculture^3,4^ and carbon stock,^5,6^ and the development of mitigation options.^7,8^ Ensemble modeling has long been used by climate modelers to overcome uncertainty in understanding processes, but it is a relatively new concept in the domain of agricultural system modeling.^9^ Running multiple biogeochemical models and model versions, in combination with different sets of site conditions, helps to distil uncertainty derived from individual model simulations.^2^ It is generally accepted by the modeling community that—provided models are diverse and independent—the prediction error decreases when using the ensemble approach.^10^ A number of questions, however, continue to prompt discussion and debate about what model ensemble studies tell us about the uncertainty surrounding the impact of the future climate on agriculture and the effectiveness of climate mitigation strategies in agriculture under different emission scenarios.^3,11,12^ As well, the use of multiple models generally increases the range of results, increases the workload, and requires more diverse skillsets to be successful.^13,14^ The answers to these questions are relevant beyond the bounds of agricultural science, as climate mitigation and adaptation decisions may be influenced by what is learned from multi-model ensemble studies.

Terrestrial biogeochemical and eco-physiological models typically comprise sets of mathematical equations simulating a continuum of interlinked atmosphere–plant–soil processes (e.g., plant photosynthesis, organic matter decomposition, ammonia volatilization, nitrification, and denitrification), enabling the simulation of spatial–temporal patterns of carbon (C) and nitrogen (N) cycles in crop and grassland systems and subsequent responses of GHG emissions to agricultural practices.^3,15−17^ As a result of their fixed, semi-empirical, and nonlinear model structure, biogeochemical models were often described as black-box models.^18,19^ They often have many parameters (e.g., 100–1000) that have no intuitive meaning^20,21^ and/or cannot be measured and must be inferred from the data. Consequently, one of the main challenges in biogeochemical modeling is that bulk observations of C and N cycling or GHG emissions rarely contain sufficient information to reliably estimate model parameters.^12^

Agricultural model intercomparison studies are becoming increasingly common. To date, a number of studies have discussed the complexity and limitations characterizing agroecosystems from multi-model ensemble studies.^3,22−25^ In model ensemble studies, there is uncertainty about the structural limitations of the model from which the contribution of agricultural systems should be generated.^26^ There is also uncertainty about how the initial conditions (i.e., input data) in the model simulations should be interpreted;^28^ uncertainty in model internal coefficients that cannot be altered by the users; and further uncertainty concerning which processes are included in the model by the developer.^20,21^ This gives rise to a branch of studies examining automatic multi-objective parameterization of several model parameters simultaneously.^13^ Ensemble studies include and compare results from models that have varying development histories, funding support, as well as varying priorities of developers, including their perceived importance of processes and parameters. Depending on the intent with which a model was built, some models include representations of agricultural processes that other models do not include, and based on the model structure, each model may require different input data and calibration strategies. Accordingly, there may be substantial variability between model outputs when different modelers are using the same calibration data, even when all are using the same model and version.^3,27,28^

There is a growing realization that the complexity of model ensemble studies arises not only due to the models used but also because of the human dimension that has a prominent role to play, considering the experience, perceptions, expectations, and approaches brought forth by modelers to calibrate parameters and validate results. The human dimension remains a key but often recalcitrant source of uncertainty.^23^ In this context, there is little information on the social and psychological aspects of model calibration or intercomparison, including how parameters are chosen for calibration, how parameters are calibrated or weighted against available data, and how models are technically verified and outputs are validated against observed data.^29^ To address this gap, we surveyed and interviewed several modelers who contributed to a model ensemble study that aimed to simulate productivity and nitrous oxide (N_2_O) emissions from cropland and grassland sites spanning four continents.^3^ These modelers varied in nationality, experience, gender, and discipline, giving us an ideal cross-section of geographical and disciplinary expertise. We analyzed the rationale used by these modelers in a multi-stage model ensemble study where different model types were compared across five successive stages (i.e., from blind parameterization to partial and full calibration) to benchmark their performance in relation to the input data provided at each stage.^3^ The objectives are to describe: (i) the heterogeneity in modelers’ prioritization of different variables in modeling decision contexts, (ii) the perceived importance of the variables across the five stages of the modeling protocol, (iii) the perceived variable structure and interrelationships, and (iv) a process through which surveys of modelers’ insights can be used to improve model intercomparison guidelines.

## Materials and Methods

The model ensemble study described in Ehrhardt et al.^3^ was based on the contribution of 24 modelers from 11 countries, reporting the results of 24 process-based integrated C–N models by comparing multi-year (1–11 years) simulations with experimental data from nine sites (four temperate permanent grassland sites and five arable crop rotations with wheat, maize, rice, and other crops). Following the multi-stage modeling protocol of Ehrhardt et al.,^3^ here, we implemented a multi-criteria decision-making (MCDM) method that collected and analyzed information on the modeling experience, priorities, and decisions made by the modelers who contributed to the model ensemble study.

### Multi-stage Modeling Protocol

**Figure 1:**
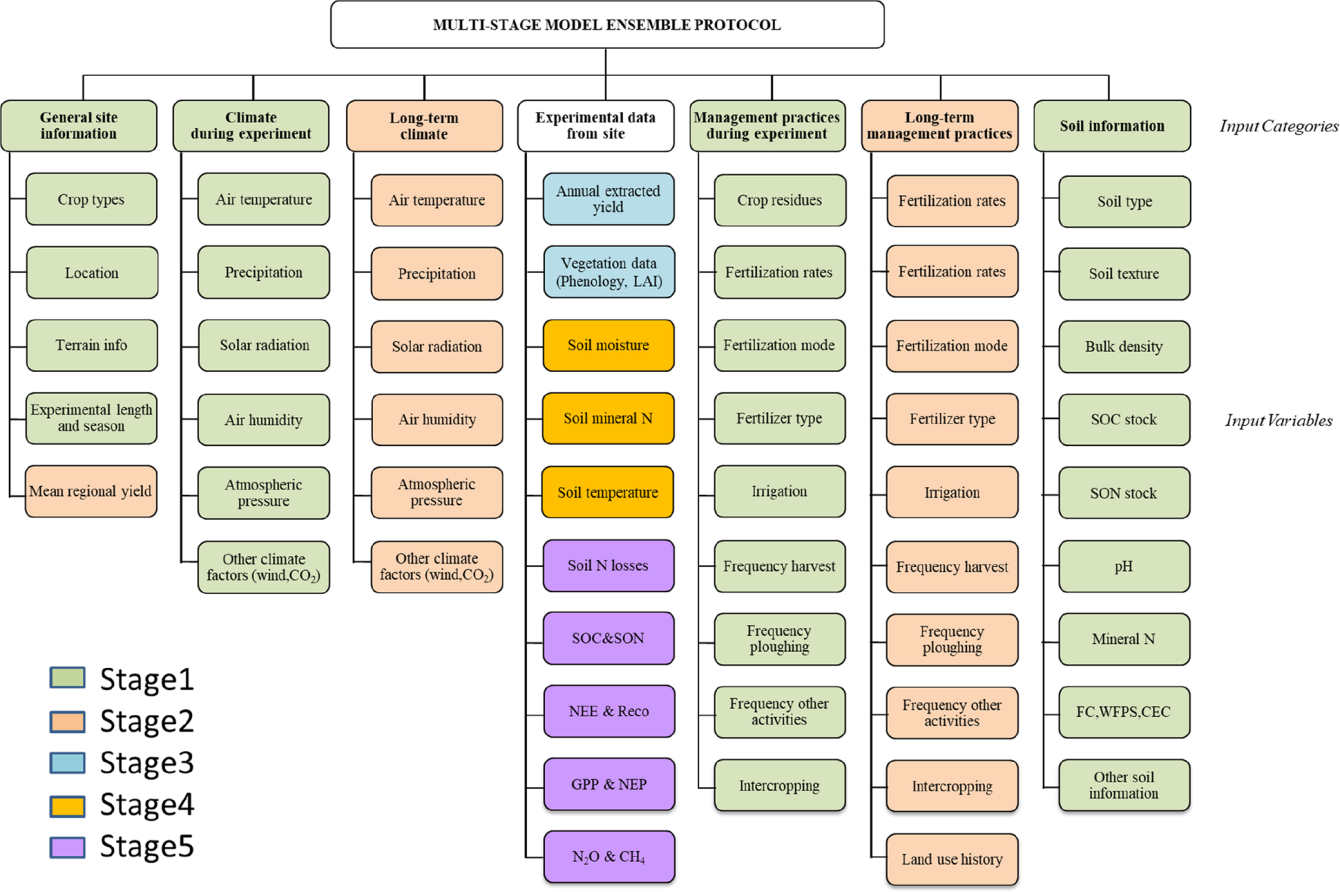
Framework of the variable partition between input categories and variables used in the five stages of the model ensemble protocol described in Ehrhardt et al. (2018).^3^

The model ensemble protocol described in Ehrhardt et al.^3^ included 55 input variables clustered into seven categories that were released to the modelers in successive stages (Figure 1). In stage 1, input data used for initial model testing included information on experimental farm site conditions [such as general site information (SI), climate during the experiment (CL), management practices during the experiment (MPDE), and soil information (SOI)]. Stage 2 provided long-term (i.e., historical) site-specific data on climate (LTCL) and management practices (LTMP) for the long-term model calibration period.^3^ Stage 3 provided part of the experimental data from site (EDS) describing plant phenology, crop/grassland vegetation development (e.g., leaf area index), and grain yields or monthly grassland offtake (biomass removed by haying or animal intake determined monthly). In stage 4, modelers accessed additional EDS data on the dynamic trends of soil temperature, moisture, and mineral N during the experiment. Finally, stage 5 included the remaining EDS information against which model outputs were compared, such as agricultural productivity (ANPP together with daily changes in live weights of livestock and daily grassland offtake), GHG emissions, and soil organic C (SOC) stock changes. In the five modeling stages, modelers were free to choose a calibration procedure of their choice based on their own subjective knowledge, the model type used, and the agricultural system targeted.

### Framework of the Survey

This study was introduced during a meeting of the Global Research Alliance on Agricultural Greenhouse Gases hosted by former INRA (currently INRAe) in Paris (France) on 13–15 December 2017. In this workshop, the modelers discussed the objectives of the survey in relation to the work performed in previous multi-stage model ensemble studies. Following this meeting, the modelers were invited to participate in the survey, which included a consent form and a background questionnaire to be completed prior to receiving the questionnaire (see S1 and S2 in the Supporting Information). In particular, the background questionnaire collected general information such as gender, education level, academic rank, modeling experience, location, institution, general features of the model/model version used, and the calibration method adopted.

A second invitation was sent to the modelers who agreed to participate in the survey, which included a participant instruction document explaining the methodology used in the survey, a demonstration video accompanied by a video help script describing how to complete the pairwise questionnaire (see S3 in the Supporting Information). The pairwise questionnaire included a number of pairwise comparison matrices (PCMs) grouped by variable categories, where the modelers assessed the relative importance and influence (i.e., relationship) that each input variable had against each other. In particular, we asked the modelers to use pre-defined rating scales to rank the data based on the steps followed during the stages of the model intercomparison study (see S4 in Supporting Information).

After completing the pairwise questionnaire, the participants received a third invitation for an interview. The interviews were conducted using telephones or videoconferences and were “semi-structured” into a list of open-ended questions (see S5 in the Supporting Information) that allowed participants to fully express their opinions on the questionnaire.^30^ Broad topics discussed with each participant included (1) feedback on the study, (2) problems encountered during the pairwise process, and (3) discussion of the pairwise results with the possibility to change any response.

### Multi-criteria Decision-Making Questionnaire

**Figure 2:**
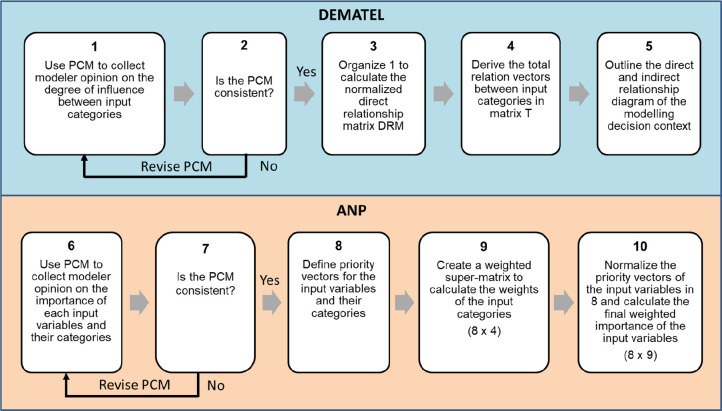
Steps of the multi-criteria decision method process combining the decision-making trial and evaluation laboratory (DEMATEL) and the analytic network process (ANP) methods. Through DEMATEL, we visualize the perceived relationship existing between different variable categories. While in ANP, the strength of the relationships outlined in DEMATEL is integrated in a network of dependencies and feedback among input variables to determine their relative importance across the five stages of the modeling protocol.

The 12 model types used in the ensemble study encompassed biogeochemical processes (e.g., plant growth, organic matter decomposition, atmospheric processes, ammonia volatilization, nitrification, denitrification, and other carbon and nitrogen processes) designed to interact with each other to describe the cycling of water, C, and N for the target ecosystems.^26^ As such, across the five modeling stages, each modeler subjectively decided how to select and prioritize the parameters that should be calibrated using the input data provided and how their model outputs should be validated against specific observed data. In particular, each modeler selected the parameters that they deemed to be the most important in contributing to high model performance (i.e., the quality of fit of several output variables to the provided data). To deal with the complexity, we applied an MCDM process (Figure 2) that combined the decision-making trial and evaluation laboratory (DEMATEL)^31^ with the analytic network process (ANP) method.^32^ Using DEMATEL, we visualized the complex interrelationships between the different variable categories, outlining the degree of influence imparted by each category, as envisaged by the modelers. In ANP, the strength of relationships outlined in DEMATEL was integrated into a network of dependencies and feedback to determine the relative importance of each input variable across the five stages of the modeling protocol (see S6 in Supporting Information).

### Data Analysis

To assess the level of agreement between the modelers, Kendall’s concordance coefficient (*K*_W_)^33^ was applied to the importance scores for the variable categories and input variables included in the pairwise questionnaires (eq 1)
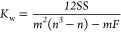
1where SS is the sum-of-squares from sums of rank scores *a*_*ij*_ (see eq 9 in S6 of the Supporting Information), *n* is the number of elements in the PCMs, *m* is the number of modelers that participated in the survey, and *F* is a correction factor for tied ranks.^34^ The null hypothesis of *K*_w_ is that the modelers provided independent ranking scores for each input variable and category (i.e., the modelers were not in agreement with each other). Perfect agreement is indicated by *K*_w_ values of 1, while no agreement is indicated by values of 0. When the null hypothesis was rejected, we tested significant effects (*p* < 0.05) against the null hypothesis that there is no agreement between the modelers.

A one-way multivariate analysis of variance was applied using SPSS statistical software (IBM SPSS v.25) to determine whether there were differences in the ratings (i.e., dependent variables) given by the modelers in the pairwise questionnaires based on the 12 model types used and their modeling experience ranging from <5 to >20 years. Wilks’ lambda test was utilized to determine whether there were significant differences (*p* < 0.05) between the mean scores of the modelers across the combination of dependent variables.

Data analysis included the correlation between the MCDM results (i.e., modeling priorities) and the ensemble modeling prediction errors described in Ehrhardt et al.^3^ Model prediction error, in particular, was represented by the root mean square error normalized by the mean of the observed data (RRMSE) of the individual models across the five stages for simulations of N_2_O emissions from arable and grassland systems; maize, wheat, and rice crop yields; and ANPP in grasslands.^3^

The relationship between RRMSE and modeling priorities across stages was investigated as
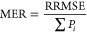
2where, ∑*P*_*i*_ represents the cumulative modeling importance of the input variable (see eq 10 in S6 of the Supporting Information) across the five stages of the model ensemble protocol, and MER is the model error rate corresponding to the change in RRMSE per unit of importance given to the input variable accessed across the five stages.

## Results and Discussion

### Characteristics of Participating Modelers

Table 1 shows an overview of the information gathered in the background questionnaire and during the interviews with the modelers who participated in the survey. Overall, the 20 modelers that participated in the study were aged between 25 and 64 years, the majority were male (54%), 68% held a PhD degree, 58% were employed under fixed-term contracts, and 84% had >5 years of modeling experience. Modelers within the 35–44 and 45–54 age categories generally used, and had published, information from a larger number of models (Table 1). The 20 modelers interviewed used 12 different model types:

i APSIM (The Agricultural Production Systems sIMulator)^35^
ii CERES-EGC (Crop Environment REsource Synthesis-Environnement et Grandes Cultures)^36^
iii DayCent and Daily DayCent^37^
iv DNDC (DeNitrification-DeComposition)^38,39^
v Landscape DNDC^40^
vi DSSAT (Decision Support System For Agro-technology Transfer)^41−43^
vii EPIC (Environmental Policy Integrated Climate)^44^
viii PaSim (Pasture Simulation model)^45^
ix DairyMod/SGS^46^
x FASSET^47^
xi STICS^48^
xii INFOCROP^49^

**Table 1:** Background Information Reported by Age Class of the Modelers Participating in the Multi-stage Intercomparison Protocol and MCDM Survey^a^

AC	N	F (%)	PhD (%)	FTC (%)	E (%)	MU	MP	MT
25–34	4	0	50	75	50	from 1 to 4	from 1 to 4	Daycent, DNDC, Manure DNDC, Century, SPA/DALEC, EU-Rotate_N, FASSET, FarmAC
35–44	7	29	100	71	86	from 1 to 7	from 1 to 4	CERES-EGC, PaSim, FarmSim, EcoSys, Armosa, Daycent, DSSAT, EPIC, APEX, ModVege, Gemini, DairyMod, APSIM, GrassGro, AusFarm, GrazFeed, SGS, FarMax
45–54	7	43	57	43	100	from 1 to 7	from 1 to 7	AusFarm, DNDC, Daycent, Century, Tier II IPCC, RZWQM2, LEACHM, InfoCrop, DSSAT, STICS, Daycent, Century, SGS, DairyMod, RothC, DairyMod, GrassGro
55–64	1	100	0	0	100	3	3	Overseer, FarMax, APSIM

a *N* = number of modelers, *F* = proportion of modelers identified as female, PhD = proportion of modelers holding a PhD degree, FTC = the proportion of modelers with a fix-term contract, MU = knowledge on the number of models, MP = number of models published in peer-reviewed articles, and MT = type of models used.

Further details are provided in the Supporting Information of Ehrhardt et al.,^3^ Appendix S1.

### Modelers’ Prioritization and Uncertainties in the Variables Provided

During the interviews, the modelers discussed their systematic approach across the five stages of the modeling protocol, as well as the uncertainties they encountered when answering the pairwise questionnaire. Here, we summarize and explain some of the uncertainties discussed with the modelers in relation to the modeling decision contexts.

In the model ensemble study, the modelers were given a set of choices about how many parameters should be calibrated against the available input data and how the models should be evaluated when the model outputs are validated against the observed data. Based on the information gathered from the interviews, in the first two stages of the modeling protocol, the modelers based their model calibration on their own experience and knowledge of the expected outcomes. In the last three stages, most modelers adopted the “trial-and-error” calibration routine, with only one modeler consistently applying Bayesian calibration. It is plausible that the gradual access to input data across the five stages negatively influenced the logic applied by the modelers in the calibration and validation processes, employing inconsistent modeling decisions between each stage (i.e., cognitive biases^50^).

The results of the pairwise questionnaires confirmed that all modelers showed some level of inconsistency in judging the relative importance of the input variables. The consistency of the modeler’s judgments was assessed through the consistency ratio (CR), which outlines the degree of bias in the pairwise judgments related to the rank order and mutual preference of alternative input data within each input category (Table 2). In this context, the responses from one modeler were excluded from the analysis due to high inconsistency (CR >30%) above the 10% cut-off threshold. The remaining 19 modelers completed the questionnaire with a consistency ratio of 7 ± 1% (mean ± standard deviation). Where the CR was above 10%, an in-person review was undertaken with the modelers to address the source of inconsistencies and find possible corrections. CR was above 10% for 37% of the modelers when ranking the variables in SOI, 21% for the scores given to EDS, 11% for the variables listed in MPDE and LTMP, and 5% when ranking the variables in SI and LTCL. Behavioral science could help to further address these findings. The pairwise judgments expressed by the modelers may have been affected by systematic biases in judgments, which reduced the complex tasks of determining the importance and influence of several input variables within each category to simpler judgmental operations related to the modeling approach. Some of these biases may be mediated by “heuristics principles” in judgments under uncertainties, overconfidence, neglect of base-rate information, and overestimates of the frequency of events that are easy to recall.^51^

**Table 2:**
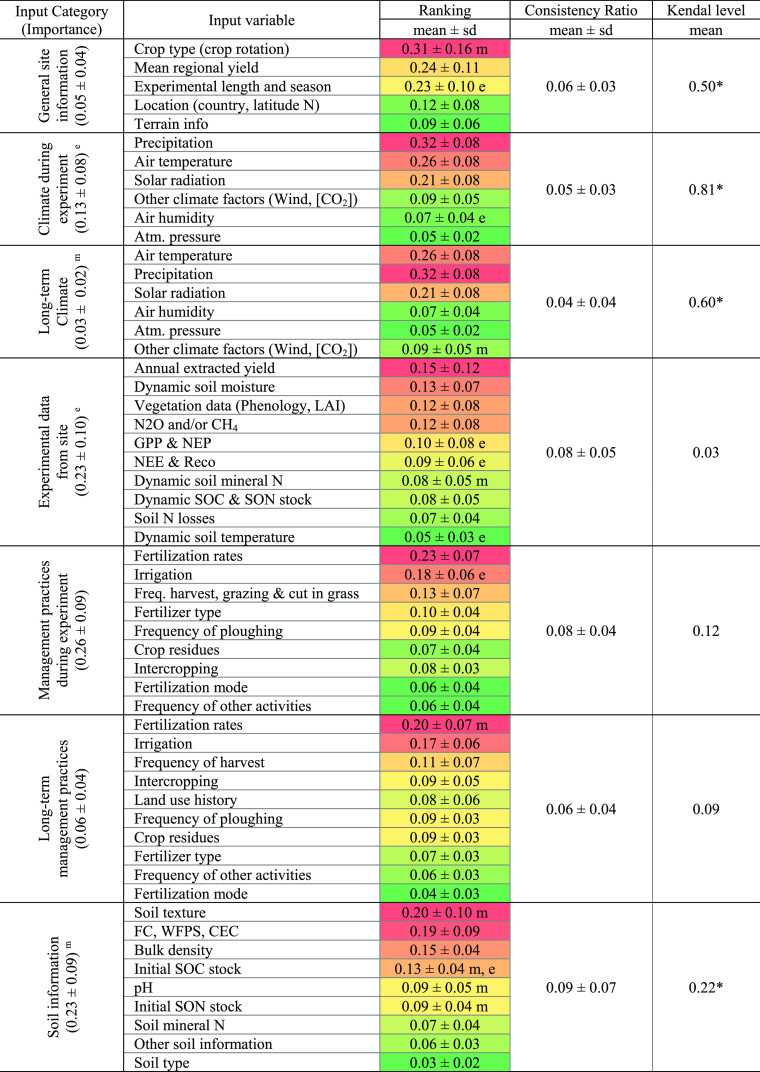
Summary of the Importance of the Input Variables and their Categories, the Consistency Ratio of the Modeler’s Judgments in the Pairwise Comparison Matrix of Each Input Category, and the Level of Kendall Concordance between the Modelers

a The ranking scores (i.e., importance) with letters *m* and *e* are significantly different at the *p* < 0.05 level between model types and modeler’s experience groups, respectively. * indicates the level of concordance significant within each variable category at the *p* < 0.05 level. Footnote: the color gradient indicates where the relative importance of each input variable falls within each variable category.

### Importance of (and Interactions between) Different Calibration Variables Perceived by Modelers

The use of DEMATEL and ANP allowed visualization of the perceived importance and the relationship between the input data across the five stages of the modeling protocol. Overall, in the ensemble study, stage 1 included more than 50% of the input variables used in the simulations (i.e., 28 input variables) (Figure 1) and accounted for 67% of importance in the model ensemble framework (Table 3). In contrast, the cumulative importance of the inputs released in stage 2 was 11%, 6% for stage 3, 5% for stage 4, and 11% for stage 5. We found a common agreement between modelers about the importance of the data used in stage 1 to initialize the models for calibration, which comprised data included in the categories SI, CL, SOI, and MPDE (Table 2). The high importance of MPDE may reflect the fact that the models involved in the ensemble study required information about farming practices such as harvesting, mowing, fertilization, tillage, and irrigation.^26^ Whereas, the low level of agreement for the priority attributed to MPDE may reflect differences in the simulations of cropland and grassland systems, as well as model characteristics, rather than disagreement between modelers on the relative importance of the input variables in MPDE. However, the importance of input variables such as the fertilization rate, irrigation regime, soil texture, field capacity and/or water-filled pore space, pH, SOC and soil organic nitrogen (SON) stocks, and atmospheric CO_2_ concentration were statistically different when classified according to model types (Table 2).

**Table 3:** Cumulative Importance of the Five Stages of the Model Ensemble Protocol^a^

input category	input variable	stage 1 (0.67 ± 0.02)	stage 2 (0.11 ± 0.01)	stage 3 (0.06 ± 0.03)	stage 4 (0.05 ± 0.01)	stage 5(0.11 ± 0.02)
soil information	soil type	0.01 ± 0.01				
	soil texture	0.05 ± 0.04				
	bulk density	0.04 ± 0.15				
	SOC stock	0.03 ± 0.13				
	SON stock	0.02 ± 0.09				
	pH	0.02 ± 0.09				
	soil mineral N	0.02 ± 0.07				
	FC, WFPS, and CEC	0.04 ± 0.19				
	other soil information	0.01 ± 0.06				
climate exp.	air temperature	0.03 ± 0.03				
	precipitation	0.04 ± 0.03				
	solar radiation	0.03 ± 0.01				
	air humidity	0.01 ± 0.01				
	atm. Pressure	0.01 ± <0.00				
	other climate factors	0.01 ± <0.00				
management practices during experiment	crop residues	0.02 ± 0.01				
	fertilization rates	0.06 ± 0.03				
	fertilization mode	0.02 ± 0.01				
	fertilizer type	0.03 ± 0.02				
	irrigation	0.05 ± 0.02				
	frequency of plowing	0.02 ± 0.01				
	frequency other activities	0.02 ± 0.01				
	intercropping	0.02 ± 0.01				
	freq. harvest, grazing, and cut in grass	0.03 ± 0.01				
general site information	crop type	0.02 ± 0.01				
	location	0.01 ± 0.01				
	terrain info	<0.00 ± <0.00				
	experimental length	0.01 ± 0.02				
	mean regional yield		0.01 ± 0.01			
long-term climate	air temperature		0.01 ± <0.00			
	precipitation		0.01 ± 0.01			
	solar radiation		0.01 ± 0.01			
	air humidity		<0.00 ± <0.00			
	atm. Pressure		<0.00 ± <0.00			
	other climate factors		<0.00 ± <0.00			
long-term management practices	fertilization rates		0.01 ± 0.01			
	fertilization mode		<0.00 ± <0.00			
	fertilizer type		<0.00 ± <0.00			
	irrigation		0.01 ± 0.01			
	frequency of harvest		0.01 ± 0.01			
	frequency of plowing		0.01 ± <0.00			
	frequency other activities		<0.01 ± <0.00			
	crop residues		0.01 ± <0.00			
	intercropping		0.01 ± <0.00			
	land use history		0.01 ± 0.01			
experimental data from site	annual extracted yield			0.03 ± 0.03		
	vegetation data (phenology, LAI)			0.03 ± 0.03		
	soil temperature				0.01 ± 0.01	
	soil moisture				0.03 ± 0.01	
	soil mineral N				0.02 ± 0.01	
	SOC and SON					0.02 ± 0.01
	GPP and NEP					0.02 ± 0.02
	NEE and Reco					0.02 ± 0.02
	soil N losses					0.02 ± 0.01
	N_2_O and/or CH_4_					0.03 ± 0.03

a Within each stage, the ranking of the input variables shown in Table 2 was normalized over the importance score of their corresponding categories. Footnote: SOC = soil organic carbon, SON = soil organic nitrogen, FC = field capacity, WFPS = water field pore space, CEC = cation exchange capacity, GPP = gross primary production, NEP = net ecosystem production, NEE = net ecosystem exchange, and Reco = ecosystem respiration.

**Figure 3:**
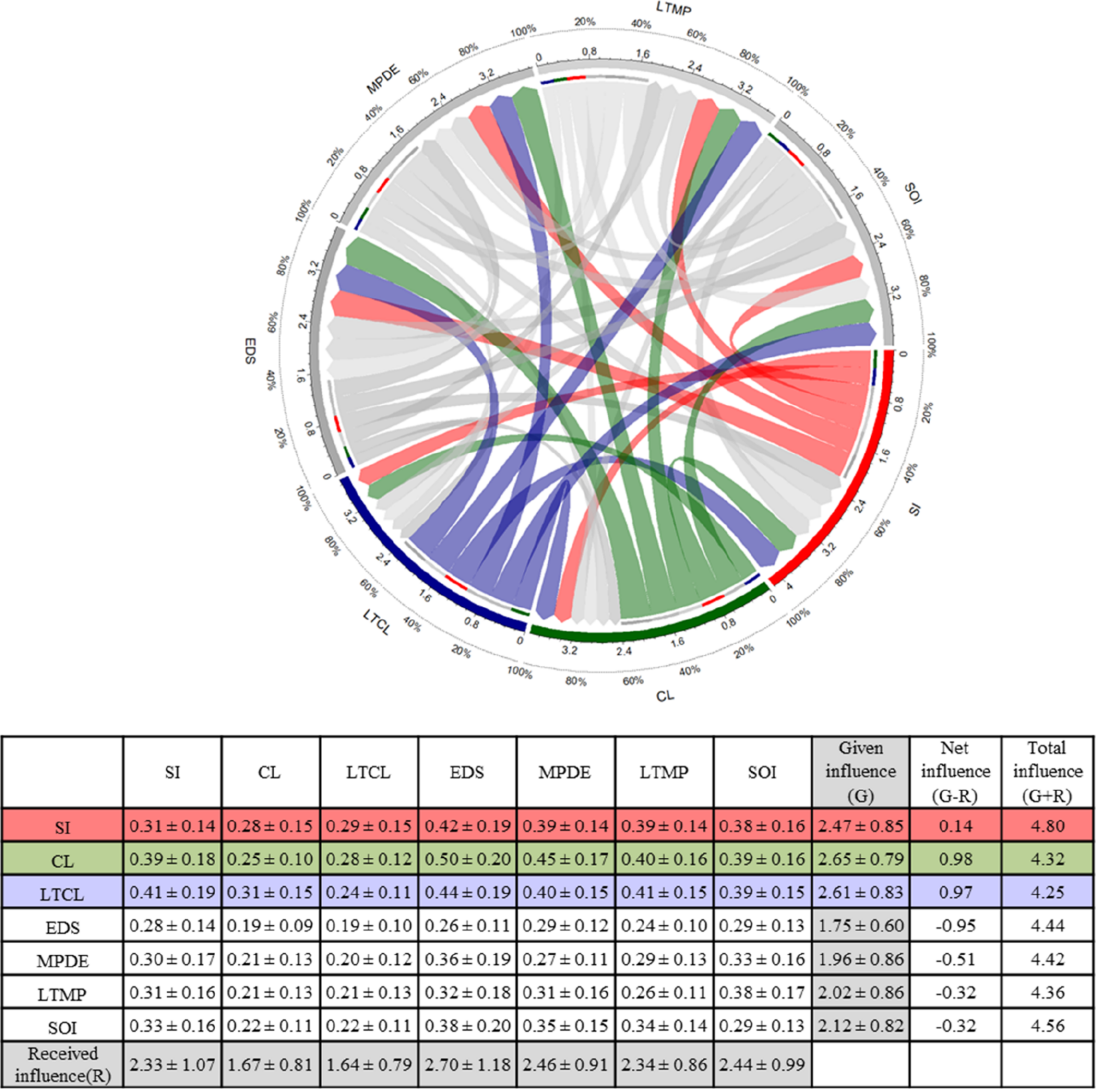
Table reports the total relation matrix of DEMATEL summarizing the level (mean ± sd) of direct and indirect influence given (G) and received (R) in each input category, the net influence (G – R), and the total level of influence (or dominance) (G + R) of the model input category used in the model ensemble study. Categories with positive G – R have a net influence toward the value of other variable categories and are denoted as “influential” categories. The circular diagram outlines the causal relationship in the model ensemble protocol between general site information (SI; red lines), climate during experiment (CL; green lines), long-term climate (LTCL; purple lines), management practices during experiments (MPDE), long-term management practices (LTMP), environmental data from site (EDS), and soil information (SOI). The arrows in the diagram show the direction and the level of influence that each input category gives and receives from other categories. The colored arrows highlight the three variable categories that resulted in being net influencers in the model ensemble protocol (i.e., positive G – R). Radial bar numbers represent the total level of influence R + C and the relative percentage of the casual relationship within each input category.

The input data given in stage 1 in the categories CL, LTCL, and SI were considered net influencers in the modeling protocol (Figure 3). This means that 60% of the relationship within the climate variables (CL and LTCL) was directed toward other input variables (i.e., a positive relationship). In contrast, the categories EDS, MPDE, LTMP, and SOI, which spread the data across the five modeling stages, were considered net receivers, with >50% of their relationship based on the influence received from other variable categories (i.e., a negative relationship). In particular, the category EDS used in stages 3, 4, and 5 (Table 3) included important in-season and end-of-season experimental data used to validate model outputs, such as site-specific experimental data on crop phenology, grassland offtake, dynamic soil processes, crop yields, ANPP, GHG emissions, and SOC stock changes. The low level of agreement between the modelers about the priorities given to EDS may reflect the heterogeneity in modelers’ knowledge on the use of experimental data for model calibration. In the model intercomparison study, the models APSIM, DairyMod, and DayCent were used by more than one modeler or modeling team. For these model types, the opinion about variables included in the categories MPDE, SOI, and EDS was characterized by low levels of agreement between modelers. The modelers that used APSIM and DairyMod, in particular, prioritized information on yield and dynamic vegetation. While, for the modelers that used DayCent, the importance of EDS was focused on parameters related to the components of the ecosystem GHG budget (such as N_2_O and CH_4_ emissions) or gross primary production (GPP), net ecosystem production (NEP), net ecosystem exchange (NEE), and ecosystem respiration (Reco) (see Table in Supporting Information S7).

Overall, the importance given to input variables such as experimental duration, GPP, NEP, NEE, Reco, and soil temperature was statistically different among modelers with different experience (Table 2). This is an important result, as the trial-and-error manual calibration routines applied in the final stage of the modeling protocol depend not only on users’ knowledge and expertise of the model structure but also on their understanding of the variables measured in the targeted agroecosystems.^52^ The analysis of the influence given and received between the variables showed contradictory results for EDS, which had a negligible influence on the value of variables included in CL, LTCL, MPDE, and SI (Figure 3). The SI category, in particular, was perceived as a net influencer and included a relatively high incoming influence in the system. Further investigation would be needed to understand whether these results are due to biases related to (i) specific features of the model structure, (ii) physical or biogeochemical processes characterizing agricultural systems, (iii) the complexity of the multi-stage modeling protocol in answering the pairwise questionnaires, or (iv) the uncertainty and variability implicit to the measured input data. In addition to the MCDM analysis, we used qualitative interviews to better understand how modelers’ attitudes (e.g., best practices), the influence of outside actors (e.g., fellow researchers, literature), and other factors (e.g., data quality, time constraints) impact their approach to modeling (manuscript in preparation).

### Relationship between Modeling Decisions and Uncertainty of the Ensemble Outcomes

**Figure 4:**
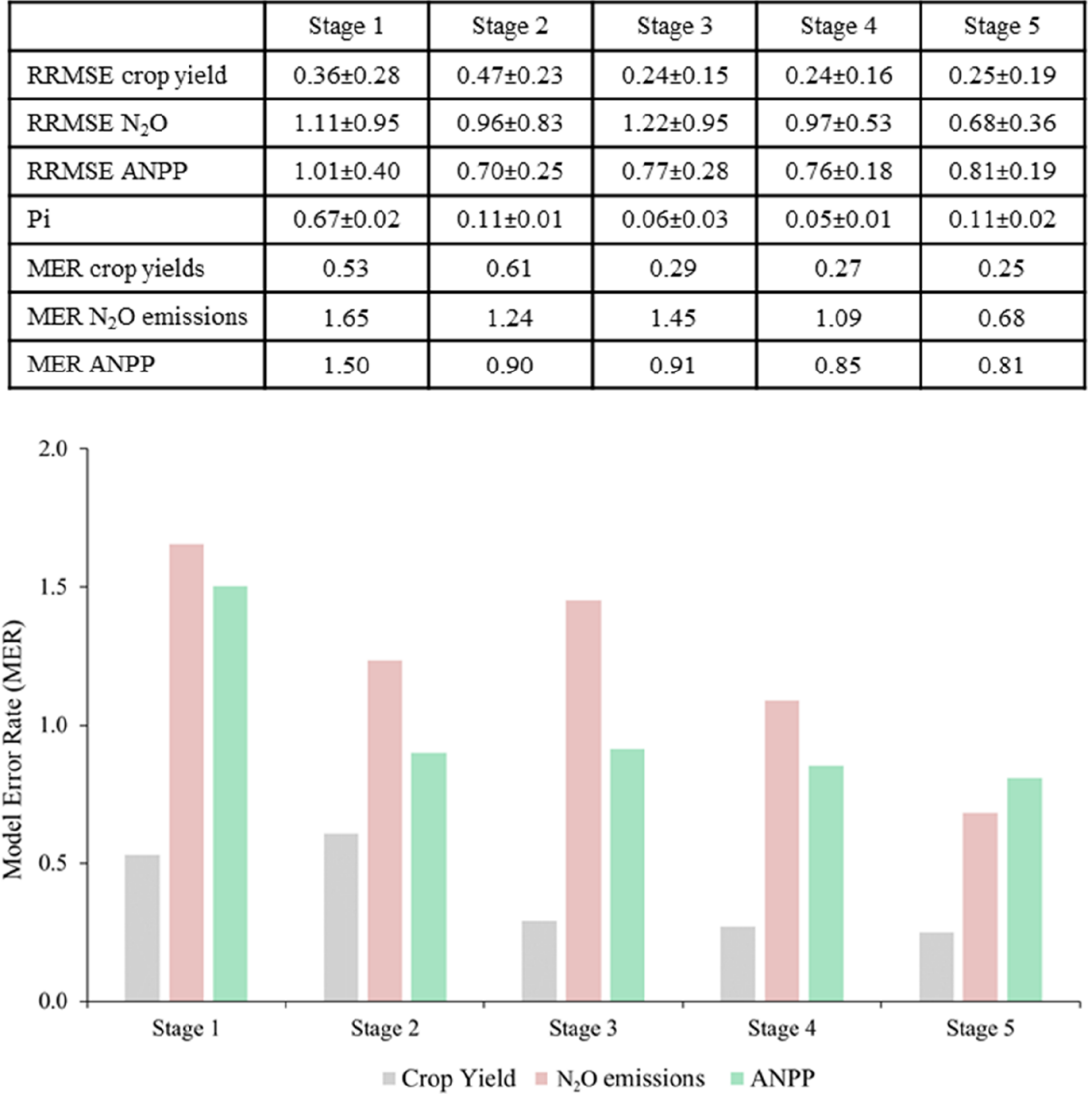
Table summarizes the relative root mean square error (RRMSE) averaged across 19 models for the ensemble simulations of soil N_2_O emissions from arable and grassland systems, crop yields of annual crop monocultures such as maize, wheat, and rice, and above-ground net primary productivity in grassland (ANPP). Pi corresponds to the cumulative modeling importance of the input variable accessed in the five stages of the model ensemble framework. MER represents the rate of model simulation error for yield, N_2_O, and ANPP per unit of modeling importance in each stage. The bar chart below the table outlines the trend of MER across the five stages of the model ensemble protocol.

Overall, the patterns of uncertainty between single models and model ensemble simulations suggest that the modeler’s choices were governed by general rational rules. However, across the five modeling stages, modelers may have come across significant challenges, particularly when the same numerical result could be arrived at in multiple ways (i.e., the right answer for the wrong reasons). In the context of decision-making, the modeler’s decision could have been restricted by “narrow framing”,^53^ limited “accessibility”, which is a technical term for the ease with which mental contents come to mind,^54^ and “decision bracketing”.^55^ The choices that the modelers faced arose one at a time, and the problems were considered as they arose. This means that in each modeling stage, the problem at hand and the immediate consequences of the choices made were far more accessible than all other considerations, and as a result, the overall modeling problem was framed far more narrowly than rational modeling assumes. In that respect, we found that the gradual access to additional input data across the five stages did not show a clear benefit in reducing the model ensemble uncertainty (Figure 4). Across the five stages, the mean RRMSE of the model simulations was 99% for N_2_O emission, 81% for ANPP, and 31% for crop yield (Figure 4). It is plausible that the gap between high model complexity and limited data availability in the initial stages of modeling generated uncertainties related to parameter equifinality or non-identifiability and ill-defined problems.^12,13,56−58^ In particular, equifinality or non-identifiability arises when different combinations of parameter values give the same results. Such results have been shown to be sensitive to the inclusion of extreme events, such as very wet and dry seasons, in the calibration.^59^ Ill-posed problems occur when the number of parameters to be optimized is greater than the boundary conditions and the number of measured data points used in model calibration.^13,20,21^

The number of input data and their perceived importance were clustered in the first two stages of the modeling study (Table 3). This limited the possibility to extract detailed information about the incremental effect of the different variable categories on ensemble simulations. The change in model prediction errors per unit of data set importance given by the modelers (MER) showed that in the crop productivity simulation, the input variables used in the first two stages (i.e., 78% of overall dataset importance) were sufficient to calibrate the models and obtain plausible results. The ensemble simulations of N_2_O emissions and ANPP, however, showed that only after receiving approximately 90% of all input data of the modeling protocol, the modelers were able to achieve the highest accuracy of the ensemble simulations. In particular, the use of historical data on climate and management practices in stage 2 reduced the MER by 25% for the ensemble prediction of N_2_O emissions in stage 1. However, in stage 3, the additional access of experimental information on vegetation data such as LAI, plant phenology, and extracted yields (i.e., 6% of the relative modeling importance) increased the MER for N_2_O emission simulation by 18%. Only with access to additional experimental data in stage 4 (dynamic measurement of soil moisture, temperature, and mineral N) did the simulation of N_2_O emissions improve, with a mean reduction in MER of 50% compared to that in stage 1. The ANPP predictions showed a similar trend in MEP as the N_2_O emissions. In this case, however, the ANPP predictions of ANPP benefited only marginally from access to site-specific experimental data in stages 3, 4, and 5 (Figure 4).

The development of generic guidelines including information about how to characterize the data required for agroecosystem modeling, with complementary and clear protocols for estimating model parameters and validating model results, remains a major challenge of agroecosystem model studies. Here, we used a multi-model ensemble study to highlight the psychology of modelers in ranking and interpreting the variables used in the simulations.

Two major conclusions can be drawn from our analysis. First, modelers perceive variables such as general site information, climate conditions, and management practices as being of vital importance for modeling cropland and grassland systems. The perceived importance of these variables was related to the calibration of processes in the first two stages of the modeling protocol, requiring information such as precipitation, air temperature, crop yield, fertilization rate, irrigation regime, soil texture, field capacity, and water-filled pore space. However, these input variables were not sufficient to obtain satisfactory ensemble simulations of crop production and GHG emissions. In this respect, the intercomparison study here showed that the crop yield simulations achieved plausible results after accessing the crop phenology and yield values, which corresponded to 84% of the variables given in the whole modeling protocol. These findings agree with ref (23), who identified minimum input data requirements for crop model intercomparisons including weather, soil, and crop management data, as well as some site-specific measurements of crop responses to test a given comparison.

Second, the framework for multi-model intercomparison studies needs to pay more attention to the structure of the models, the understanding of the interrelationships between the different processes, and the experience of the modelers. The models used in the ensemble study included numerous biogeochemical processes (e.g., plant growth, organic matter decomposition, atmospheric processes, ammonia volatilization, nitrification, and denitrification) designed to interact with each other to describe the water, C, and N cycles for the target ecosystems.^28^ In this context, we visualized the relationship between the different variables used in a multi-stage modeling protocol, partitioning them into the categories of net influencers and net receivers. Although general site information and climate data only represent 30% of the input data used in the ensemble protocol, the modelers’ opinions on the importance and level of influence of these variables used to initialize the model calibrations depended on the model type used. In addition, the ensemble simulations of N_2_O emissions and grassland above-ground biomass required more than 90% of the input data used in the modeling protocol (i.e., four out of five stages) to obtain plausible results. In this context, Ehrhardt et al.^3^ outlined several limitations in the calibration methods and model structures that could explain the discrepancies between simulated and observed data. The opinion of the modelers, however, was that fundamental parameters such as crop management, soil characteristics, and experimental data from sites were net receivers in the framework of the modeling protocol. Importantly, the ranking of the most important input data, such as experimental length and season, irrigation, SOC stock, soil temperature, GPP, NEP, NEE, and Reco, varied according to the experience of the modelers. We argue that it is likely that among the limitations explaining the uncertainty of the ensemble study, the interpretation made in the “trial-and-error” calibration routines and the structure of the modeling protocol itself also lead to uncertainty in the simulations. What is natural and intuitive in a given modeling situation is not the same for everyone: different experiences favor different modeling intuitions about the meaning of input variables, and modeling behaviors become intuitive as skills are acquired.^51^ In the Ehrhardt et al.^3^ study, only one modeling team used the automatic calibration method. It is plausible that in automatic calibration methods, the selection of parametrization algorithm or software is one such human decision factor among many that could have a large bearing on the validity of calibration and consequential model performance. Thus, the experience and skills of the modelers again influence model outputs via their initial capability, knowledge, and confidence in using a given approach for calibration.

Moving forward, ensemble studies should include in their guidelines an understanding of how data interpretations and model structures influence the calibration and validation strategies and collect information on this. This study would have been particularly helpful if it had been carried out before and during the model ensemble study, as the information obtained could have contributed to the guidelines for the ensemble study. The structure of the multi-stage benchmarking protocol was a major limitation of our analysis. First, the model intercomparison study involved 20 modelers that used 12 distinct model types. This means that in our study, only for three model types did we have the possibility to sample more than the modeler. Second, the first two stages of the protocol comprised the majority of the input data used by the modelers, corresponding to 78% of the variables considered by the modelers to be the most important. In this context, a release of data across the stages in line with modeling priorities and model structures could have helped to organize the five stages of the ensemble study to understand the relative contribution between data interpretation, model calibration methods, model structures, and site-specific variability of observations to the uncertainty of the ensemble simulation.

## Supporting Information Available

Modeler’s survey consent form and a background questionnaire to be completed prior to receiving the participant instruction document; two pairwise questionnaires; open-ended questions to be answered prior to the telephonic or videoconference interview; multi-criteria decision-making methodology, and summary of the opinion of the modelers that used the models APSIM, DairyMod, and DayCent (PDF)
