# Supplementary material for: How Modelers Model: the Overlooked Social and Human Dimensions in Model Intercomparison Studies
Source: Environ Sci Technol. 2022 Sep 2;56(18):13485–98. doi: 10.1021/acs.est.2c02023 (PMC9494747; doi:10.1021/acs.est.2c02023)
Supplement: Supplementary file 1 — es2c02023_si_001.pdf [file es2c02023_si_001.pdf]

Supporting Information *for*

How modellers model: the overlooked social and human dimensions in model intercomparison studies

Fabrizio Albanito[1]*, David McBey[1]*, Matthew Harrison[2], Pete Smith[1], Fiona Ehrhardt[3,4], Arti Bhatia[5], Gianni Bellocchi[6], Lorenzo Brilli[7], Marco Carozzi[8], Karen Christie[9], Jordi Doltra[9], Christopher Dorich[10], Luca Doro[11,12], Peter Grace[13], Brian Grant[14], Joël Léonard[15], Mark Liebig[16], Cameron Ludemann[17], Raphael Martin[6], Elizabeth Meier[18], Rachelle Meyer[19], Massimiliano De Antoni Migliorati[13, 20], Vasileios Myrgiotis[21], Sylvie Recous[22], Renáta Sándor[23], Val Snow[24], Jean-François Soussana[3], Ward N. Smith[14] and Nuala Fitton[1]

[1]University of Aberdeen, Institute of Biological and Environmental Sciences, School of Biological Science, 23 St. Machar Drive, AB24 3UU, Aberdeen, UK
[2]Tasmanian Institute of Agriculture, University of Tasmania, Newnham Drive, Launceston, TAS, 7248 Australia
[9]Tasmanian Institute of Agriculture, University of Tasmania. 16-20 Mooreville Rd, Burnie, TAS, 7320 Australia
[3]INRAE, CODIR, 75007 Paris, France
[4]RITTMO AgroEnvironnement, , 68000 Colmar, France
[5]ICAR-Indian Agricultural Research Institute, New Delhi, 110012, India
[6]Université Clermont Auvergne, INRAE, VetAgro Sup, UREP, 63000 Clermont-Ferrand, France
[7]CNR-IBE, National Research Council Institute for the BioEconomy, Via Caproni 8, 50145, Florence, Italy
[8]UMR ECOSYS, INRAE, AgroParisTech, Université Paris-Saclay, 78850, Thiverval-Grignon, France
[10]Sustainable Field Crops Programme, Institute of Agrifood Research and Technology (IRTA) Mas Badia, 17134 La Tallada d'Empordà, Girona, Spain
[11]Natural Resource Ecology Lab, Colorado State University, Fort Collins, CO, 80521 USA
[12]Texas A&M AgriLife Research, Blackland Research and Extension Center, Temple, Texas 76502, USA
[13]Desertification Research Centre, University of Sassari, 07100 Sassari, Italy
[14]Queensland University of Technology, Brisbane, Queensland 4000, Australia
[15]Ottawa Research and Development Centre, Agriculture and Agri-Food Canada, Ottawa, ON, K1A 0C6, Canada
[16]BioEcoAgro Joint Research Unit, INRAE, 02000 Barenton-Bugny, France
[17]USDA-ARS Northern Great Plains Research Laboratory, P.O. Box 459, Mandan, ND, 58554 USA
[18]Cameron Ludemann Consulting, Arnhem, The Netherlands, 6821 EV
[19]CSIRO Agriculture and Food, St Lucia, Qld 4067, Australia
[20]University of Melbourne, Faculty of Veterinary & Agricultural Sciences, Parkville, VIC 3010, Australia
[21]Department of Environment and Science, Dutton Park, Queensland 4102, Australia
[22]School of GeoSciences, University of Edinburgh, EH9 3JN, Edinburgh, UK
[23]Université de Reims Champagne-Ardenne, INRAE, FARE laboratory, 51100 Reims, France
[24]Agricultural Institute, Centre for Agricultural Research, ELKH, 2462 Martonvásár, Hungary
[25]AgResearch, PB 4749, Christchurch 8140, New Zealand

This supporting Information includes 22 pages, 14 figures and 2 tables

S1. Consent form

Title of Study: How modellers model

Please read this form carefully. Once you have read each statement please tick the relevant box to acknowledge your consent. Please then print your name, sign and date the form.

- ☐ I have fully read the accompanying Participation information sheet and consent to my answers* being used in any publication as part of the larger study
- ☐ I am willing for the researchers to contact me again with any follow up questions that maybe required
- ☐ I confirm that I have read and understood the particpant information sheet for the above study. I have had the opportunity to consider the information, ask questions and have had those answered satisfactorily
- ☐ I understand that my particpation is voluntary and that I am completely free to withdraw from the study or any part of the study at any time I wish
- ☐ I understand that data collected from me may be used for research into environmental modellers behaviour. These data may be looked at by responsible indiviuals from the University of Aberdeen or regulatory bodies
- ☐ I agree to take part in the above study
- ☐ I agree that this workbook is not for distribution to third parties

Name of participant:
Signature:
Date:
Email address:

on [Date]

[Place]

[Name]

[email address]

Signature

Thank you in advance for taking part in the survey and if you have any questions please do not hesitate to contact one of the research team:
Dr. Nuala Fitton (n.fitton@abdn.ac.uk), Dr. Fabrizio Albanito (f.albanif@abdn.ac.uk) or Dr. Dave McBey (d.mcbey@abdn.ac.uk).

*please note an individuals answer will only be published after being made anonymous

S2. Participant background questionnaire

How Modellers Model: Questionnaire

This section is comprised of a number of general questions on your background. Please answer honestly and do not reference any specific work or papers that could be used to identify you.

1. Model Code:
2. Gender:
3. Age:
4. Degree and PhD qualification:
5. Current position, please detail if you are permanent, fixed term contract or temporary:
6. Number of years with modelling experience:
7. Number and names of models you typically use:
8. Number and names of models you have published with [based on your use of the model]:
9. Greenhouse gases you typically simulate:
10. Greenhouse gases you have published with work based on your modelling results:
11. What method would most accurately describe you calibration approach? (i) trial and error, (ii) Bayesian calibration, (iii) Generalised likelihood uncertainty estimation (GLUE) or (iv) Other [please detail]:
12. Did you find that your calibration approach changed as more data became available in the five stages:

Further information:

If you require any questions or require any further information please contact one of the research team: Dr. Nuala Fitton (n.fitton@abdn.ac.uk), Dr. Fabrizio Albanito (f.albanif@abdn.ac.uk) or Dr. Dave McBey (d.mcbey@abdn.ac.uk) or Fiona Ehrhardt (fiona.ehrhardt@gmail.com)

S3. Participant instruction document

How Modellers Model

As a participant in the JPI-FACCE model inter-comparison, you are receiving this document as you have previously agreed to participate in this study. Details on how to complete the different sections are given below, however please do not hesitate to contact us if you have any questions.

WHAT DO I HAVE TO DO?

This study consists of: (i) a questionnaire (word document titled: General information), (ii) a pairwise comparisons of model input factors and categories [excel file titled; Decision making analysis – Pairwise survey].

Participants are asked to read and complete each section carefully prior to starting the different sections of the survey. For consistency purposes, participants are asked to take time when answering questions and if necessary take a break from the survey for completion later. Answers may also be changed **at any time** prior to submission and each section provides the possibility for participants to provide comments supporting their answers if required.

Upon submission all answers will be stored in accordance with best practice and only available to the study research team. We ask that participants submit answers as honestly as possible and to facilitate this each respondents answers will be anonymised, treated without prejudice, and will not be shared with third parties or companies. Finally, as participation is on a volunteer basis submitted answers can be withdrawn at any time prior to any publication.

(i) Questionnaire

This section is comprised of a number of general questions on the participant's background. The questions can be found and answered in the accompanying word document titled "Questionnaire".

(ii) Pairwise comparison of model input factors

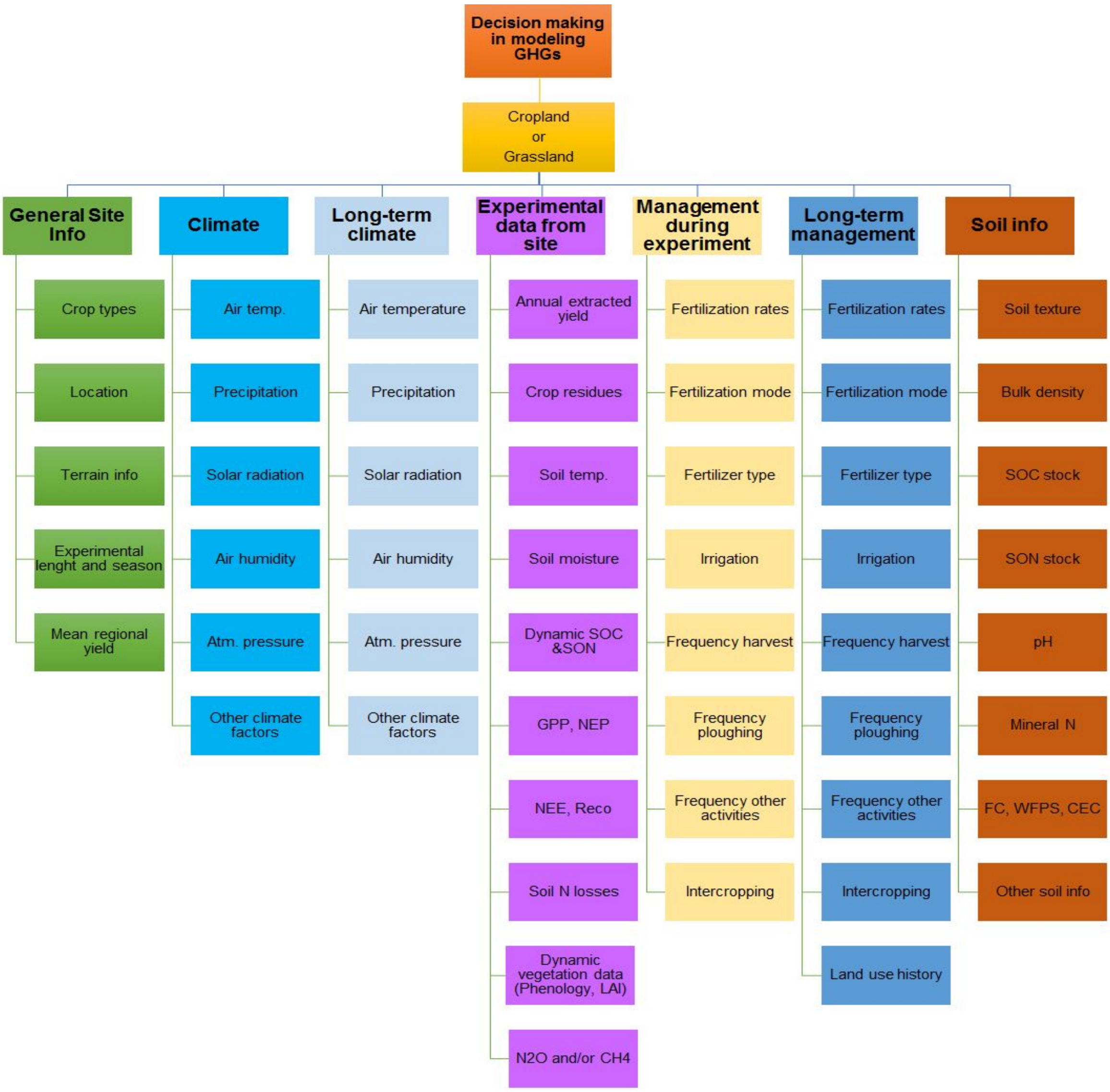

Figure S1: Schematic diagram detailing the hierarchical order of the ecosystems, input categories and inputs used in the pairwise comparison

Pairwise Priorities

In environmental modelling decision making is in general based on prioritizing a number of input parameters used across distinct processes of the model. These input parameters must be interpreted for meaning and usefulness according to their priority to serve specific model processes and environmental circumstances, the decision of which is left to the modeller. These decisions may become a complex problem when the pertinent input parameters are expressed in different units or, because of the nature of the model process, their relative importance is difficult to quantify. Consequently these decisions become intangible factors actively contributing to the final results that need to be quantified.

Therefore, the aim of this section is to use the pairwise comparison to evaluate the relative importance of each input parameter, as well as the overall importance of categories in your final model decision making.

Here, in the excel file titled: Decision making analysis – Pairwise survey (see Supplementary Information S4), participants will find the input parameters organized into different sections distinguished by specific parameter categories i.e. site information, climate, soil, long term or short-term experimental data, etc. In tab 1 of the accompanying excel file titled "Pairwise – priorities" participants will be asked to complete the pairwise comparisons of the categories used in the model inter-comparison study and then for the specific inputs within each category (Figure S1). Participants, should detail the ***importance*** that each pair of categories and specific data inputs may have against each other. Values will be reported using the scale of absolute judgement reported in Figure S2 below, and participants are requested to judge the relative importance or priority of each input parameter in their modelling decisions. The judgment scale represents how much more one parameter dominates another parameter with respect to a given category and model process component.

Comparisons should be made for each row individually and where a participant thinks that the category listed on the left is more important than that on the right, they should select the appropriate box on the left hand scale. Similarly if the participant thinks that the category/input listed on the right is more important the appropriate box along the scale on the right hand side should be ticked. Where the inputs are deemed to be of equal importance please select 1. Where compromises, or more refined considerations, are needed between two adjacent judgments the participants can use the intermediate values. **Participants are kindly asked to answer the pairwise sections keeping in mind the GHG's and ecosystem/ecosystems you submitted results for in the inter-comparison.**

Intensity of importance	*Definition*	*Explanation*
1	Equal importance	Two factors contribute equally to the objective
3	Moderate important	Experience and judgement slightly favour one factor over another
5	Strong importnce	Experience and judgement strongly favour one factor over another
7	Very strong importance	Experience and judgement very strongly favour one factor over another
9	Extreme importance	The evidende of favouring one factor over another is of the highest possible order of affirmation
2,4,6,8	Intermediate values	

Figure S2: Judgement scale to use in the pairwise comparisons found in the Pairwise Priorities tab

As a guide to completing this section, participants will find a progress bar can be found on the top right hand - side of the Pairwise Priorities tab and to the right of each pairwise section (Figure S3).

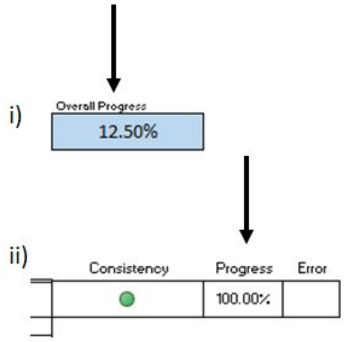

Figure S3: (i) Overall progress bar and (ii) section progress bar; both are found in the Pairwise Priorities tab

As stated previously, participants are asked to take their time when answering the pairwise section of the survey. To ensure that your answers remain consistent throughout the different sections of the pairwise comparison participants will find on the right hand side of each section a consistency indicator (Figure S4). Here a green light in the cell indicates that good level of consistency, yellow indicates a consistency ratio level below the optimal level and a red light indicates an unacceptable level of

consistency. The traffic lights provide the result of a consistency check of the opinion expressed in the different pairwise comparisons.

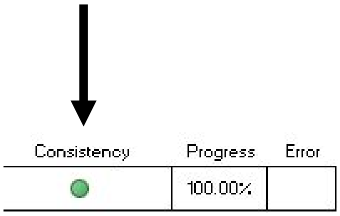

Figure S4: Consistency indicator found in the Pairwise Priorities tab

Typically in studies that use this methodology, participants who take careful consideration of their answers tend to have a good consistency ratio. Therefore for this section **we kindly ask that this section is completed taking time and consideration in all the answers**. In instances of a red light we would advise a review of the judgements selected across the section.

Finally, the error cell will indicate, via a yellow flag (Figure S5), if there is an error within the table for example more than one judgement made per line.

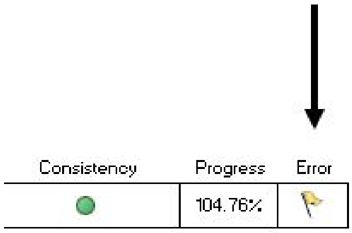

Figure S5: Error indicator found in the Pairwise Priorities tab

Pairwise – Influences

While the previous section focused on the importance of different categories/inputs we know that in biogeochemical models these are interconnected. Thus forming a complex structure that must be accounted for in your decision making. The following pairwise aims to understand the degree of influences that each category of input type, as defined in the inter-comparison, might have on other categories.

This pairwise questionnaire is found in tab 2 of the excel file and is titled Pairwise – Influences. Participants are asked to fill in the pairwise questionnaire of the categories judging the degree of ***influence*** that each category may have on the other categories (Figure S6). To complete this section please carefully examine Figure 1, which details the input parameters that define each category and answers should be based on these.

Judgement scale to use in the pariwise comparisons of the broad categories		
Degree of Influence	*Definition*	*Explanation*
0	No	When a category has no influence on another one
1	Low	When a category has low influence on another one
2	Moderate	When a category has moderate influence on another one
3	High	When a category has high influence on another one
4	Very High	When a category has very high influence on another one

Figure S6: Judgement scale to use in the pairwise comparisons found in the Pairwise influences tab

Within the pairwise itself, each line of the pairwise should be answered twice in relation to the categories listed in column A and B. For example on the left hand side please indicate the degree of influence category "General site Information" has on the category "Climate", then on the right hand

side please detail the influence the category "Climate" has on "General Site Information". Please note also that if you think that one category has no influence on the other select zero and if neither category has an influence on each other tick just the zero box in the pair-wise line. Answers should be based on using the judgement scale detailed in table 2, once each line has been completed correctly a green flag with text "OK" will appear (Figure S7). **Answers may be changed at any time prior to submission and we kindly request that participants take their time when making each comparison.**

PAIRWISE QUESTIONARRIE OF CATEGORIES

CATEGORY A	More Influence				No Influence	More Influence				CATEGORY B	Progress
	4	3	2	1	0	1	2	3	4		
General site Information	☐	☐	☐	☑	☐	☐	☐	☐	☑	Climate	OK

Figure S7: Progress indicator found in the Pairwise Influences tab

RETURNING THE COMPLETED FILES AND NEXT STAGE:

Participants are asked to read this instruction document carefully to ensure that they understand the study then complete and sign the accompanying consent form. Once completed participants are asked to return all the completed documents; consent form, questionnaire and pairwise files; to Fiona Ehrhardt, via email fiona.ehrhardt@gmail.com by **1st of March 2018.**

Files are to be saved according to the following format: please note that the model code here will be the same as the one you used in the inter-comparison study

1. **Consent form_MODELCODE_X.X**
2. **Questionaire_MODELCODE_X.X**
3. **Decision making analysis – Pairwise survey_ MODELCODE_X.X**

Once submissions have been received and reviewed participants will then be contacted to arrange a SKYPE interview on a time and date that suits the participant. This should take approximately 1 hour and should be centered on gathering the participants on a range of topics that are relevant to the modelling community and follow up on their answers given in the pairwise survey. The interview will be recorded and conducted by Dr. Dave McBey, in addition any transcription made of answers given will also be anonymized.

Further information

If you require any questions or require any further information please contact one of the research team: Dr. Nuala Fitton (n.fitton@abdn.ac.uk), Dr. Fabrizio Albanito (f.albanif@abdn.ac.uk) or Dr. Dave McBey (d.mcbey@abdn.ac.uk) or Fiona Ehrhardt (fiona.ehrhardt@gmail.com)

References:

Ehrhardt, F., et al, Assessing uncertainties in crop and pasture ensemble model simulations of productivity and N_2O emissions. Global Change Biology, 2017, doi: 10.1111/gcb.13965

S4. Pairwise questionnaires

This section includes two pairwise questionnaries used to judge the importance of the input variables within each categories, and the relative influence that each category might have on other categories, respectively. The first questionnarie is divided into 8 pairwise comparison matrices (PCMs) (from page S9 to page S14). The second questionnarie includes one PCM at page S15.

Using the judgement scale below, please fill in the 8 pairwise questionnaires judging the importance that each pair categories and specific data inputs may have against each other. Select only one judgement value per line. The "Consistency", "Progress", and "Error" cells on the right of the tables highlight the correct completion of the questionnaire. A green light in the Consistency cell highlights good level of consistency, a yellow light highlights a consistency ratio just below optimal level, and a red light highlights a level of consistency unacceptable. In case of red light we suggest to review the judgements selected across the table. The cell progress provides the percentage of completion of the table, and a yellow flag in the Error cell highlights potential error in the table (e.g. more than one judgement value selected per pairwise).

PLEASE FILL IN THE QUESTIONNARIE BELOW REFERRING TO THE ECOSYSTEMS AND GHGs YOU SUBMITTED IN THE INTER-COMPARISON

Table 1: Judgement scale to use in the pariwise comparisons

Intensity of importance	*Definition*	*Explanation*
1	Equal importance	Two factors contribute equally to the objective
3	Moderate important	Experience and judgement slightly favour one factor over another
5	Strong importnce	Experience and judgement strongly favour one factor over another
7	Very strong importance	Experience and judgement very strongly favour one factor over another
9	Extreme importance	The evidende of favouring one factor over another is of the highest possible order of affirmation
2,4,6,8	Intermediate values	

Overall Progress
#REF!

Data Categories

1	CATEGORY A	More Important								Equal	More Important								CATEGORY B
		9	8	7	6	5	4	3	2	1	2	3	4	5	6	7	8	9	
	General site Information	☐	☐	☐	☐	☐	☐	☐	☐	☐	☐	☐	☐	☐	☐	☐	☐	☐	Climate
	General site Information	☐	☐	☐	☐	☐	☐	☐	☐	☐	☐	☐	☐	☐	☐	☐	☐	☐	Long-term Climate
	General site Information	☐	☐	☐	☐	☐	☐	☐	☐	☐	☐	☐	☐	☐	☐	☐	☐	☐	Experimental Data From Site
	General site Information	☐	☐	☐	☐	☐	☐	☐	☐	☐	☐	☐	☐	☐	☐	☐	☐	☐	Management Practices During Experiment
	General site Information	☐	☐	☐	☐	☐	☐	☐	☐	☐	☐	☐	☐	☐	☐	☐	☐	☐	Long-Term Management Practices
	General site Information	☐	☐	☐	☐	☐	☐	☐	☐	☐	☐	☐	☐	☐	☐	☐	☐	☐	Soil Information
	Climate	☐	☐	☐	☐	☐	☐	☐	☐	☐	☐	☐	☐	☐	☐	☐	☐	☐	Long-term Climate
	Climate	☐	☐	☐	☐	☐	☐	☐	☐	☐	☐	☐	☐	☐	☐	☐	☐	☐	Experimental Data From Site
	Climate	☐	☐	☐	☐	☐	☐	☐	☐	☐	☐	☐	☐	☐	☐	☐	☐	☐	Management Practices During Experiment
	Climate	☐	☐	☐	☐	☐	☐	☐	☐	☐	☐	☐	☐	☐	☐	☐	☐	☐	Long-Term Management Practices
	Climate	☐	☐	☐	☐	☐	☐	☐	☐	☐	☐	☐	☐	☐	☐	☐	☐	☐	Soil Information
	Long-term Climate	☐	☐	☐	☐	☐	☐	☐	☐	☐	☐	☐	☐	☐	☐	☐	☐	☐	Experimental Data From Site
	Long-term Climate	☐	☐	☐	☐	☐	☐	☐	☐	☐	☐	☐	☐	☐	☐	☐	☐	☐	Management Practices During Experiment
	Long-term Climate	☐	☐	☐	☐	☐	☐	☐	☐	☐	☐	☐	☐	☐	☐	☐	☐	☐	Long-Term Management Practices
	Long-term Climate	☐	☐	☐	☐	☐	☐	☐	☐	☐	☐	☐	☐	☐	☐	☐	☐	☐	Soil Information
	Experimental Data From Site	☐	☐	☐	☐	☐	☐	☐	☐	☐	☐	☐	☐	☐	☐	☐	☐	☐	Management Practices During Experiment
	Experimental Data From Site	☐	☐	☐	☐	☐	☐	☐	☐	☐	☐	☐	☐	☐	☐	☐	☐	☐	Long-Term Management Practices
	Experimental Data From Site	☐	☐	☐	☐	☐	☐	☐	☐	☐	☐	☐	☐	☐	☐	☐	☐	☐	Soil Information
	Management Practices During Experiment	☐	☐	☐	☐	☐	☐	☐	☐	☐	☐	☐	☐	☐	☐	☐	☐	☐	Long-Term Management Practices
	Management Practices During Experiment	☐	☐	☐	☐	☐	☐	☐	☐	☐	☐	☐	☐	☐	☐	☐	☐	☐	Soil Information
	Long-Term Management Practices	☐	☐	☐	☐	☐	☐	☐	☐	☐	☐	☐	☐	☐	☐	☐	☐	☐	Soil Information

Consistency	Progress	Error
#N/A	0.00%	

General site Information

2	INPUT A	More Important								Equal	More Important								INPUT B
		9	8	7	6	5	4	3	2	1	2	3	4	5	6	7	8	9	
	Crop Type (crop rotation)	☐	☐	☐	☐	☐	☐	☐	☐	☐	☐	☐	☐	☐	☐	☐	☐	☐	Location (country, latitude N)
	Crop Type (crop rotation)	☐	☐	☐	☐	☐	☐	☐	☐	☐	☐	☐	☐	☐	☐	☐	☐	☐	Terrain info
	Crop Type (crop rotation)	☐	☐	☐	☐	☐	☐	☐	☐	☐	☐	☐	☐	☐	☐	☐	☐	☐	Experimental length and season
	Crop Type (crop rotation)	☐	☐	☐	☐	☐	☐	☐	☐	☐	☐	☐	☐	☐	☐	☐	☐	☐	Mean regional yield
	Location (country, latitude N)	☐	☐	☐	☐	☐	☐	☐	☐	☐	☐	☐	☐	☐	☐	☐	☐	☐	Terrain info
	Location (country, latitude N)	☐	☐	☐	☐	☐	☐	☐	☐	☐	☐	☐	☐	☐	☐	☐	☐	☐	Experimental length and season
	Location (country, latitude N)	☐	☐	☐	☐	☐	☐	☐	☐	☐	☐	☐	☐	☐	☐	☐	☐	☐	Mean regional yield
	Terrain info	☐	☐	☐	☐	☐	☐	☐	☐	☐	☐	☐	☐	☐	☐	☐	☐	☐	Experimental length and season
	Terrain info	☐	☐	☐	☐	☐	☐	☐	☐	☐	☐	☐	☐	☐	☐	☐	☐	☐	Mean regional yield (wheat or grassland)
	Experimental length and season	☐	☐	☐	☐	☐	☐	☐	☐	☐	☐	☐	☐	☐	☐	☐	☐	☐	Mean regional yield (wheat or grassland)

Consistency	Progress	Error
#N/A	0.00%	

Climate During Experiment

Consistency	Progress	Error
#N/A	0.00%	

3																		
INPUT A	More Important								Equal	More Important								INPUT B
	9	8	7	6	5	4	3	2	1	2	3	4	5	6	7	8	9	
Air temperature	☐	☐	☐	☐	☐	☐	☐	☐	☐	☐	☐	☐	☐	☐	☐	☐	☐	Precipitation
Air temperature	☐	☐	☐	☐	☐	☐	☐	☐	☐	☐	☐	☐	☐	☐	☐	☐	☐	Solar radiation
Air temperature	☐	☐	☐	☐	☐	☐	☐	☐	☐	☐	☐	☐	☐	☐	☐	☐	☐	Air humidity
Air temperature	☐	☐	☐	☐	☐	☐	☐	☐	☐	☐	☐	☐	☐	☐	☐	☐	☐	Atm. pressure
Air temperature	☐	☐	☐	☐	☐	☐	☐	☐	☐	☐	☐	☐	☐	☐	☐	☐	☐	Other climate factors (Wind sp., [CO2], Vapore pr.)
Precipitation	☐	☐	☐	☐	☐	☐	☐	☐	☐	☐	☐	☐	☐	☐	☐	☐	☐	Solar radiation
Precipitation	☐	☐	☐	☐	☐	☐	☐	☐	☐	☐	☐	☐	☐	☐	☐	☐	☐	Air humidity
Precipitation	☐	☐	☐	☐	☐	☐	☐	☐	☐	☐	☐	☐	☐	☐	☐	☐	☐	Atm. pressure
Precipitation	☐	☐	☐	☐	☐	☐	☐	☐	☐	☐	☐	☐	☐	☐	☐	☐	☐	Other climate factors (Wind sp., [CO2], Vapore pr.)
Solar radiation	☐	☐	☐	☐	☐	☐	☐	☐	☐	☐	☐	☐	☐	☐	☐	☐	☐	Air humidity
Solar radiation	☐	☐	☐	☐	☐	☐	☐	☐	☐	☐	☐	☐	☐	☐	☐	☐	☐	Atm. pressure
Solar radiation	☐	☐	☐	☐	☐	☐	☐	☐	☐	☐	☐	☐	☐	☐	☐	☐	☐	Other climate factors (Wind sp., [CO2], Vapore pr.)
Air humidity	☐	☐	☐	☐	☐	☐	☐	☐	☐	☐	☐	☐	☐	☐	☐	☐	☐	Atm. pressure
Air humidity	☐	☐	☐	☐	☐	☐	☐	☐	☐	☐	☐	☐	☐	☐	☐	☐	☐	Other climate factors (Wind sp., [CO2], Vapore pr.)
Atm. pressure	☐	☐	☐	☐	☐	☐	☐	☐	☐	☐	☐	☐	☐	☐	☐	☐	☐	Other climate factors (Wind sp., [CO2], Vapore pr.)

Overall Progress

0.00%

Long-term Climate

Consistency	Progress	Error
#N/A	0.00%	

4																		
INPUT A	More Important								Equal	More Important								INPUT B
	9	8	7	6	5	4	3	2	1	2	3	4	5	6	7	8	9	
Air temperature	☐	☐	☐	☐	☐	☐	☐	☐	☐	☐	☐	☐	☐	☐	☐	☐	☐	Precipitation
Air temperature	☐	☐	☐	☐	☐	☐	☐	☐	☐	☐	☐	☐	☐	☐	☐	☐	☐	Solar radiation
Air temperature	☐	☐	☐	☐	☐	☐	☐	☐	☐	☐	☐	☐	☐	☐	☐	☐	☐	Air humidity
Air temperature	☐	☐	☐	☐	☐	☐	☐	☐	☐	☐	☐	☐	☐	☐	☐	☐	☐	Atm. pressure
Air temperature	☐	☐	☐	☐	☐	☐	☐	☐	☐	☐	☐	☐	☐	☐	☐	☐	☐	Other climate factors (Wind sp., [CO2], Vapore pr.)
Precipitation	☐	☐	☐	☐	☐	☐	☐	☐	☐	☐	☐	☐	☐	☐	☐	☐	☐	Solar radiation
Precipitation	☐	☐	☐	☐	☐	☐	☐	☐	☐	☐	☐	☐	☐	☐	☐	☐	☐	Air humidity
Precipitation	☐	☐	☐	☐	☐	☐	☐	☐	☐	☐	☐	☐	☐	☐	☐	☐	☐	Atm. pressure
Precipitation	☐	☐	☐	☐	☐	☐	☐	☐	☐	☐	☐	☐	☐	☐	☐	☐	☐	Other climate factors (Wind sp., [CO2], Vapore pr.)
Solar radiation	☐	☐	☐	☐	☐	☐	☐	☐	☐	☐	☐	☐	☐	☐	☐	☐	☐	Air humidity
Solar radiation	☐	☐	☐	☐	☐	☐	☐	☐	☐	☐	☐	☐	☐	☐	☐	☐	☐	Atm. pressure
Solar radiation	☐	☐	☐	☐	☐	☐	☐	☐	☐	☐	☐	☐	☐	☐	☐	☐	☐	Other climate factors (Wind sp., [CO2], Vapore pr.)
Air humidity	☐	☐	☐	☐	☐	☐	☐	☐	☐	☐	☐	☐	☐	☐	☐	☐	☐	Atm. pressure
Air humidity	☐	☐	☐	☐	☐	☐	☐	☐	☐	☐	☐	☐	☐	☐	☐	☐	☐	Other climate factors (Wind sp., [CO2], Vapore pr.)
Atm. pressure	☐	☐	☐	☐	☐	☐	☐	☐	☐	☐	☐	☐	☐	☐	☐	☐	☐	Other climate factors (Wind sp., [CO2], Vapore pr.)

Experimental Data From Site

Consistency	Progress	Error
#N/A	0.00%	

5 INPUT A	More Important								Equal	More Important								INPUT B
	9	8	7	6	5	4	3	2	1	2	3	4	5	6	7	8	9	
Annual extracted grain yield	☐	☐	☐	☐	☐	☐	☐	☐	☐	☐	☐	☐	☐	☐	☐	☐	☐	Dynamic Soil Temperature
Annual extracted grain yield	☐	☐	☐	☐	☐	☐	☐	☐	☐	☐	☐	☐	☐	☐	☐	☐	☐	Dynamic Soil Moisture
Annual extracted grain yield	☐	☐	☐	☐	☐	☐	☐	☐	☐	☐	☐	☐	☐	☐	☐	☐	☐	Dynamic Soil Mineral N
Annual extracted grain yield	☐	☐	☐	☐	☐	☐	☐	☐	☐	☐	☐	☐	☐	☐	☐	☐	☐	Dynamic SOC & SON
Annual extracted grain yield	☐	☐	☐	☐	☐	☐	☐	☐	☐	☐	☐	☐	☐	☐	☐	☐	☐	GPP & NEP
Annual extracted grain yield	☐	☐	☐	☐	☐	☐	☐	☐	☐	☐	☐	☐	☐	☐	☐	☐	☐	NEE & Reco
Annual extracted grain yield	☐	☐	☐	☐	☐	☐	☐	☐	☐	☐	☐	☐	☐	☐	☐	☐	☐	Soil N losses
Annual extracted grain yield	☐	☐	☐	☐	☐	☐	☐	☐	☐	☐	☐	☐	☐	☐	☐	☐	☐	Dynamic vegetation data (Phenology, LAI)
Annual extracted grain yield	☐	☐	☐	☐	☐	☐	☐	☐	☐	☐	☐	☐	☐	☐	☐	☐	☐	N2O and/or CH4
Dynamic Soil Temperature	☐	☐	☐	☐	☐	☐	☐	☐	☐	☐	☐	☐	☐	☐	☐	☐	☐	Dynamic Soil Moisture
Dynamic Soil Temperature	☐	☐	☐	☐	☐	☐	☐	☐	☐	☐	☐	☐	☐	☐	☐	☐	☐	Dynamic Soil Mineral N
Dynamic Soil Temperature	☐	☐	☐	☐	☐	☐	☐	☐	☐	☐	☐	☐	☐	☐	☐	☐	☐	Dynamic SOC & SON
Dynamic Soil Temperature	☐	☐	☐	☐	☐	☐	☐	☐	☐	☐	☐	☐	☐	☐	☐	☐	☐	GPP & NEP
Dynamic Soil Temperature	☐	☐	☐	☐	☐	☐	☐	☐	☐	☐	☐	☐	☐	☐	☐	☐	☐	NEE & Reco
Dynamic Soil Temperature	☐	☐	☐	☐	☐	☐	☐	☐	☐	☐	☐	☐	☐	☐	☐	☐	☐	Soil N losses
Dynamic Soil Temperature	☐	☐	☐	☐	☐	☐	☐	☐	☐	☐	☐	☐	☐	☐	☐	☐	☐	Dynamic vegetation data (Phenology, LAI)
Dynamic Soil Temperature	☐	☐	☐	☐	☐	☐	☐	☐	☐	☐	☐	☐	☐	☐	☐	☐	☐	N2O and/or CH4
Dynamic Soil Moisture	☐	☐	☐	☐	☐	☐	☐	☐	☐	☐	☐	☐	☐	☐	☐	☐	☐	Dynamic Soil Mineral N
Dynamic Soil Moisture	☐	☐	☐	☐	☐	☐	☐	☐	☐	☐	☐	☐	☐	☐	☐	☐	☐	Dynamic SOC & SON
Dynamic Soil Moisture	☐	☐	☐	☐	☐	☐	☐	☐	☐	☐	☐	☐	☐	☐	☐	☐	☐	GPP & NEP
Dynamic Soil Moisture	☐	☐	☐	☐	☐	☐	☐	☐	☐	☐	☐	☐	☐	☐	☐	☐	☐	NEE & Reco
Dynamic Soil Moisture	☐	☐	☐	☐	☐	☐	☐	☐	☐	☐	☐	☐	☐	☐	☐	☐	☐	Soil N losses
Dynamic Soil Moisture	☐	☐	☐	☐	☐	☐	☐	☐	☐	☐	☐	☐	☐	☐	☐	☐	☐	Dynamic vegetation data (Phenology, LAI)
Dynamic Soil Moisture	☐	☐	☐	☐	☐	☐	☐	☐	☐	☐	☐	☐	☐	☐	☐	☐	☐	N2O and/or CH4
Dynamic Soil Mineral N	☐	☐	☐	☐	☐	☐	☐	☐	☐	☐	☐	☐	☐	☐	☐	☐	☐	Dynamic SOC & SON
Dynamic Soil Mineral N	☐	☐	☐	☐	☐	☐	☐	☐	☐	☐	☐	☐	☐	☐	☐	☐	☐	GPP & NEP
Dynamic Soil Mineral N	☐	☐	☐	☐	☐	☐	☐	☐	☐	☐	☐	☐	☐	☐	☐	☐	☐	NEE & Reco
Dynamic Soil Mineral N	☐	☐	☐	☐	☐	☐	☐	☐	☐	☐	☐	☐	☐	☐	☐	☐	☐	Soil N losses
Dynamic Soil Mineral N	☐	☐	☐	☐	☐	☐	☐	☐	☐	☐	☐	☐	☐	☐	☐	☐	☐	Dynamic vegetation data (Phenology, LAI)
Dynamic Soil Mineral N	☐	☐	☐	☐	☐	☐	☐	☐	☐	☐	☐	☐	☐	☐	☐	☐	☐	N2O and/or CH4
Dynamic SOC & SON	☐	☐	☐	☐	☐	☐	☐	☐	☐	☐	☐	☐	☐	☐	☐	☐	☐	GPP & NEP
Dynamic SOC & SON	☐	☐	☐	☐	☐	☐	☐	☐	☐	☐	☐	☐	☐	☐	☐	☐	☐	NEE & Reco
Dynamic SOC & SON	☐	☐	☐	☐	☐	☐	☐	☐	☐	☐	☐	☐	☐	☐	☐	☐	☐	Soil N losses
Dynamic SOC & SON	☐	☐	☐	☐	☐	☐	☐	☐	☐	☐	☐	☐	☐	☐	☐	☐	☐	Dynamic vegetation data (Phenology, LAI)
Dynamic SOC & SON	☐	☐	☐	☐	☐	☐	☐	☐	☐	☐	☐	☐	☐	☐	☐	☐	☐	N2O and/or CH4
GPP & NEP	☐	☐	☐	☐	☐	☐	☐	☐	☐	☐	☐	☐	☐	☐	☐	☐	☐	NEE & Reco
GPP & NEP	☐	☐	☐	☐	☐	☐	☐	☐	☐	☐	☐	☐	☐	☐	☐	☐	☐	Soil N losses
GPP & NEP	☐	☐	☐	☐	☐	☐	☐	☐	☐	☐	☐	☐	☐	☐	☐	☐	☐	Dynamic vegetation data (Phenology, LAI)
GPP & NEP	☐	☐	☐	☐	☐	☐	☐	☐	☐	☐	☐	☐	☐	☐	☐	☐	☐	N2O and/or CH4
NEE & Reco	☐	☐	☐	☐	☐	☐	☐	☐	☐	☐	☐	☐	☐	☐	☐	☐	☐	Soil N losses
NEE & Reco	☐	☐	☐	☐	☐	☐	☐	☐	☐	☐	☐	☐	☐	☐	☐	☐	☐	Dynamic vegetation data (Phenology, LAI)
NEE & Reco	☐	☐	☐	☐	☐	☐	☐	☐	☐	☐	☐	☐	☐	☐	☐	☐	☐	N2O and/or CH4
Soil N losses	☐	☐	☐	☐	☐	☐	☐	☐	☐	☐	☐	☐	☐	☐	☐	☐	☐	Dynamic vegetation data (Phenology, LAI)
Soil N losses	☐	☐	☐	☐	☐	☐	☐	☐	☐	☐	☐	☐	☐	☐	☐	☐	☐	N2O and/or CH4
Dynamic vegetation data (Phenology, LAI)	☐	☐	☐	☐	☐	☐	☐	☐	☐	☐	☐	☐	☐	☐	☐	☐	☐	N2O and/or CH4

Overall Progress

0.00%

Management Practices During Experiment

	Consistency	Progress	Error
6	#N/A	0.00%	

INPUT A	More Important								Equal	More Important								INPUT B
	9	8	7	6	5	4	3	2	1	2	3	4	5	6	7	8	9	
Crop residues	☐	☐	☐	☐	☐	☐	☐	☐	☐	☐	☐	☐	☐	☐	☐	☐	☐	Fertilization rates
Crop residues	☐	☐	☐	☐	☐	☐	☐	☐	☐	☐	☐	☐	☐	☐	☐	☐	☐	Fertilization mode
Crop residues	☐	☐	☐	☐	☐	☐	☐	☐	☐	☐	☐	☐	☐	☐	☐	☐	☐	Fertilizer type
Crop residues	☐	☐	☐	☐	☐	☐	☐	☐	☐	☐	☐	☐	☐	☐	☐	☐	☐	Irrigation
Crop residues	☐	☐	☐	☐	☐	☐	☐	☐	☐	☐	☐	☐	☐	☐	☐	☐	☐	Frequency of ploughing
Crop residues	☐	☐	☐	☐	☐	☐	☐	☐	☐	☐	☐	☐	☐	☐	☐	☐	☐	Frequency of other activities
Crop residues	☐	☐	☐	☐	☐	☐	☐	☐	☐	☐	☐	☐	☐	☐	☐	☐	☐	Intercropping
Crop residues	☐	☐	☐	☐	☐	☐	☐	☐	☐	☐	☐	☐	☐	☐	☐	☐	☐	Frequency of harvest in crop, or Frequency of grazing events or biomass cut in grass
Fertilization rates	☐	☐	☐	☐	☐	☐	☐	☐	☐	☐	☐	☐	☐	☐	☐	☐	☐	Fertilization mode
Fertilization rates	☐	☐	☐	☐	☐	☐	☐	☐	☐	☐	☐	☐	☐	☐	☐	☐	☐	Fertilizer type
Fertilization rates	☐	☐	☐	☐	☐	☐	☐	☐	☐	☐	☐	☐	☐	☐	☐	☐	☐	Irrigation
Fertilization rates	☐	☐	☐	☐	☐	☐	☐	☐	☐	☐	☐	☐	☐	☐	☐	☐	☐	Frequency of ploughing
Fertilization rates	☐	☐	☐	☐	☐	☐	☐	☐	☐	☐	☐	☐	☐	☐	☐	☐	☐	Frequency of other activities
Fertilization rates	☐	☐	☐	☐	☐	☐	☐	☐	☐	☐	☐	☐	☐	☐	☐	☐	☐	Intercropping
Fertilization rates	☐	☐	☐	☐	☐	☐	☐	☐	☐	☐	☐	☐	☐	☐	☐	☐	☐	Frequency of harvest in crop, or Frequency of grazing events or biomass cut in grass
Fertilization mode	☐	☐	☐	☐	☐	☐	☐	☐	☐	☐	☐	☐	☐	☐	☐	☐	☐	Fertilizer type
Fertilization mode	☐	☐	☐	☐	☐	☐	☐	☐	☐	☐	☐	☐	☐	☐	☐	☐	☐	Irrigation
Fertilization mode	☐	☐	☐	☐	☐	☐	☐	☐	☐	☐	☐	☐	☐	☐	☐	☐	☐	Frequency of ploughing
Fertilization mode	☐	☐	☐	☐	☐	☐	☐	☐	☐	☐	☐	☐	☐	☐	☐	☐	☐	Frequency of other activities
Fertilization mode	☐	☐	☐	☐	☐	☐	☐	☐	☐	☐	☐	☐	☐	☐	☐	☐	☐	Intercropping
Fertilization mode	☐	☐	☐	☐	☐	☐	☐	☐	☐	☐	☐	☐	☐	☐	☐	☐	☐	Frequency of harvest in crop, or Frequency of grazing events or biomass cut in grass
Fertilizer type	☐	☐	☐	☐	☐	☐	☐	☐	☐	☐	☐	☐	☐	☐	☐	☐	☐	Irrigation
Fertilizer type	☐	☐	☐	☐	☐	☐	☐	☐	☐	☐	☐	☐	☐	☐	☐	☐	☐	Frequency of ploughing
Fertilizer type	☐	☐	☐	☐	☐	☐	☐	☐	☐	☐	☐	☐	☐	☐	☐	☐	☐	Frequency of other activities
Fertilizer type	☐	☐	☐	☐	☐	☐	☐	☐	☐	☐	☐	☐	☐	☐	☐	☐	☐	Intercropping
Fertilizer type	☐	☐	☐	☐	☐	☐	☐	☐	☐	☐	☐	☐	☐	☐	☐	☐	☐	Frequency of harvest in crop, or Frequency of grazing events or biomass cut in grass
Irrigation	☐	☐	☐	☐	☐	☐	☐	☐	☐	☐	☐	☐	☐	☐	☐	☐	☐	Frequency of ploughing
Irrigation	☐	☐	☐	☐	☐	☐	☐	☐	☐	☐	☐	☐	☐	☐	☐	☐	☐	Frequency of other activities
Irrigation	☐	☐	☐	☐	☐	☐	☐	☐	☐	☐	☐	☐	☐	☐	☐	☐	☐	Intercropping
Irrigation	☐	☐	☐	☐	☐	☐	☐	☐	☐	☐	☐	☐	☐	☐	☐	☐	☐	Frequency of harvest in crop, or Frequency of grazing events or biomass cut in grass
Frequency of ploughing	☐	☐	☐	☐	☐	☐	☐	☐	☐	☐	☐	☐	☐	☐	☐	☐	☐	Frequency of other activities
Frequency of ploughing	☐	☐	☐	☐	☐	☐	☐	☐	☐	☐	☐	☐	☐	☐	☐	☐	☐	Intercropping
Frequency of ploughing	☐	☐	☐	☐	☐	☐	☐	☐	☐	☐	☐	☐	☐	☐	☐	☐	☐	Frequency of harvest in crop, or Frequency of grazing events or biomass cut in grass
Frequency of other activities	☐	☐	☐	☐	☐	☐	☐	☐	☐	☐	☐	☐	☐	☐	☐	☐	☐	Intercropping
Frequency of other activities	☐	☐	☐	☐	☐	☐	☐	☐	☐	☐	☐	☐	☐	☐	☐	☐	☐	Frequency of harvest in crop, or Frequency of grazing events or biomass cut in grass
Intercropping	☐	☐	☐	☐	☐	☐	☐	☐	☐	☐	☐	☐	☐	☐	☐	☐	☐	Frequency of harvest in crop, or Frequency of grazing events or biomass cut in grass

Overall Progress

0.00%

Long-term Management Practices

7	Consistency	Progress	Error
	**#N/A**	0.00%	

INPUT A	More Important								Equal	More Important								INPUT B
	9	8	7	6	5	4	3	2	1	2	3	4	5	6	7	8	9	
Fertilization rates	☐	☐	☐	☐	☐	☐	☐	☐	☐	☐	☐	☐	☐	☐	☐	☐	☐	Fertilization mode
Fertilization rates	☐	☐	☐	☐	☐	☐	☐	☐	☐	☐	☐	☐	☐	☐	☐	☐	☐	Fertilizer type
Fertilization rates	☐	☐	☐	☐	☐	☐	☐	☐	☐	☐	☐	☐	☐	☐	☐	☐	☐	Irrigation
Fertilization rates	☐	☐	☐	☐	☐	☐	☐	☐	☐	☐	☐	☐	☐	☐	☐	☐	☐	Frequency of harvest
Fertilization rates	☐	☐	☐	☐	☐	☐	☐	☐	☐	☐	☐	☐	☐	☐	☐	☐	☐	Frequency of ploughing
Fertilization rates	☐	☐	☐	☐	☐	☐	☐	☐	☐	☐	☐	☐	☐	☐	☐	☐	☐	Frquency of other activities
Fertilization rates	☐	☐	☐	☐	☐	☐	☐	☐	☐	☐	☐	☐	☐	☐	☐	☐	☐	Crop residues
Fertilization rates	☐	☐	☐	☐	☐	☐	☐	☐	☐	☐	☐	☐	☐	☐	☐	☐	☐	Intercropping
Fertilization rates	☐	☐	☐	☐	☐	☐	☐	☐	☐	☐	☐	☐	☐	☐	☐	☐	☐	Land use history
Fertilization mode	☐	☐	☐	☐	☐	☐	☐	☐	☐	☐	☐	☐	☐	☐	☐	☐	☐	Fertilizer type
Fertilization mode	☐	☐	☐	☐	☐	☐	☐	☐	☐	☐	☐	☐	☐	☐	☐	☐	☐	Irrigation
Fertilization mode	☐	☐	☐	☐	☐	☐	☐	☐	☐	☐	☐	☐	☐	☐	☐	☐	☐	Frequency of harvest
Fertilization mode	☐	☐	☐	☐	☐	☐	☐	☐	☐	☐	☐	☐	☐	☐	☐	☐	☐	Frequency f ploughing
Fertilization mode	☐	☐	☐	☐	☐	☐	☐	☐	☐	☐	☐	☐	☐	☐	☐	☐	☐	Frquency of other activities
Fertilization mode	☐	☐	☐	☐	☐	☐	☐	☐	☐	☐	☐	☐	☐	☐	☐	☐	☐	Crop residues
Fertilization mode	☐	☐	☐	☐	☐	☐	☐	☐	☐	☐	☐	☐	☐	☐	☐	☐	☐	Intercropping
Fertilization mode	☐	☐	☐	☐	☐	☐	☐	☐	☐	☐	☐	☐	☐	☐	☐	☐	☐	Land use history
Fertilizer type	☐	☐	☐	☐	☐	☐	☐	☐	☐	☐	☐	☐	☐	☐	☐	☐	☐	Irrigation
Fertilizer type	☐	☐	☐	☐	☐	☐	☐	☐	☐	☐	☐	☐	☐	☐	☐	☐	☐	Frequency of harvest
Fertilizer type	☐	☐	☐	☐	☐	☐	☐	☐	☐	☐	☐	☐	☐	☐	☐	☐	☐	Frequency f ploughing
Fertilizer type	☐	☐	☐	☐	☐	☐	☐	☐	☐	☐	☐	☐	☐	☐	☐	☐	☐	Frquency of other activities
Fertilizer type	☐	☐	☐	☐	☐	☐	☐	☐	☐	☐	☐	☐	☐	☐	☐	☐	☐	Crop residues
Fertilizer type	☐	☐	☐	☐	☐	☐	☐	☐	☐	☐	☐	☐	☐	☐	☐	☐	☐	Intercropping
Fertilizer type	☐	☐	☐	☐	☐	☐	☐	☐	☐	☐	☐	☐	☐	☐	☐	☐	☐	Land use history
Irrigation	☐	☐	☐	☐	☐	☐	☐	☐	☐	☐	☐	☐	☐	☐	☐	☐	☐	Frequency of harvest
Irrigation	☐	☐	☐	☐	☐	☐	☐	☐	☐	☐	☐	☐	☐	☐	☐	☐	☐	Frequency f ploughing
Irrigation	☐	☐	☐	☐	☐	☐	☐	☐	☐	☐	☐	☐	☐	☐	☐	☐	☐	Frquency of other activities
Irrigation	☐	☐	☐	☐	☐	☐	☐	☐	☐	☐	☐	☐	☐	☐	☐	☐	☐	Crop residues
Irrigation	☐	☐	☐	☐	☐	☐	☐	☐	☐	☐	☐	☐	☐	☐	☐	☐	☐	Intercropping
Irrigation	☐	☐	☐	☐	☐	☐	☐	☐	☐	☐	☐	☐	☐	☐	☐	☐	☐	Land use history
Frequency of harvest	☐	☐	☐	☐	☐	☐	☐	☐	☐	☐	☐	☐	☐	☐	☐	☐	☐	Frequency f ploughing
Frequency of harvest	☐	☐	☐	☐	☐	☐	☐	☐	☐	☐	☐	☐	☐	☐	☐	☐	☐	Frquency of other activities
Frequency of harvest	☐	☐	☐	☐	☐	☐	☐	☐	☐	☐	☐	☐	☐	☐	☐	☐	☐	Crop residues
Frequency of harvest	☐	☐	☐	☐	☐	☐	☐	☐	☐	☐	☐	☐	☐	☐	☐	☐	☐	Intercropping
Frequency of harvest	☐	☐	☐	☐	☐	☐	☐	☐	☐	☐	☐	☐	☐	☐	☐	☐	☐	Land use history
Frequency of Ploughing	☐	☐	☐	☐	☐	☐	☐	☐	☐	☐	☐	☐	☐	☐	☐	☐	☐	Frquency of other activities
Frequency of Ploughing	☐	☐	☐	☐	☐	☐	☐	☐	☐	☐	☐	☐	☐	☐	☐	☐	☐	Crop residues
Frequency of Ploughing	☐	☐	☐	☐	☐	☐	☐	☐	☐	☐	☐	☐	☐	☐	☐	☐	☐	Intercropping
Frequency of Ploughing	☐	☐	☐	☐	☐	☐	☐	☐	☐	☐	☐	☐	☐	☐	☐	☐	☐	Land use history
Frequency of other activities	☐	☐	☐	☐	☐	☐	☐	☐	☐	☐	☐	☐	☐	☐	☐	☐	☐	Crop residues
Frequency of other activities	☐	☐	☐	☐	☐	☐	☐	☐	☐	☐	☐	☐	☐	☐	☐	☐	☐	Intercropping
Frequency of other activities	☐	☐	☐	☐	☐	☐	☐	☐	☐	☐	☐	☐	☐	☐	☐	☐	☐	Land use history
Crop residues	☐	☐	☐	☐	☐	☐	☐	☐	☐	☐	☐	☐	☐	☐	☐	☐	☐	Intercropping
Crop residues	☐	☐	☐	☐	☐	☐	☐	☐	☐	☐	☐	☐	☐	☐	☐	☐	☐	Land use history
Intercropping	☐	☐	☐	☐	☐	☐	☐	☐	☐	☐	☐	☐	☐	☐	☐	☐	☐	Land use history

Overall Progress

0.00%

Specific Soil Information

8

INPUT A	More Important								Equal	More Important								INPUT B
	9	8	7	6	5	4	3	2	1	2	3	4	5	6	7	8	9	
Soil type	☐	☐	☐	☐	☐	☐	☐	☐	☐	☐	☐	☐	☐	☐	☐	☐	☐	Soil texture
Soil type	☐	☐	☐	☐	☐	☐	☐	☐	☐	☐	☐	☐	☐	☐	☐	☐	☐	Bulk density
Soil type	☐	☐	☐	☐	☐	☐	☐	☐	☐	☐	☐	☐	☐	☐	☐	☐	☐	initial SOC stock
Soil type	☐	☐	☐	☐	☐	☐	☐	☐	☐	☐	☐	☐	☐	☐	☐	☐	☐	initial SON stock
Soil type	☐	☐	☐	☐	☐	☐	☐	☐	☐	☐	☐	☐	☐	☐	☐	☐	☐	pH
Soil type	☐	☐	☐	☐	☐	☐	☐	☐	☐	☐	☐	☐	☐	☐	☐	☐	☐	initial Mineral N
Soil type	☐	☐	☐	☐	☐	☐	☐	☐	☐	☐	☐	☐	☐	☐	☐	☐	☐	FC, WFPS, CEC
Soil type	☐	☐	☐	☐	☐	☐	☐	☐	☐	☐	☐	☐	☐	☐	☐	☐	☐	Other soil information
Soil texture	☐	☐	☐	☐	☐	☐	☐	☐	☐	☐	☐	☐	☐	☐	☐	☐	☐	Bulk density
Soil texture	☐	☐	☐	☐	☐	☐	☐	☐	☐	☐	☐	☐	☐	☐	☐	☐	☐	initial SOC stock
Soil texture	☐	☐	☐	☐	☐	☐	☐	☐	☐	☐	☐	☐	☐	☐	☐	☐	☐	initial SON stock
Soil texture	☐	☐	☐	☐	☐	☐	☐	☐	☐	☐	☐	☐	☐	☐	☐	☐	☐	pH
Soil texture	☐	☐	☐	☐	☐	☐	☐	☐	☐	☐	☐	☐	☐	☐	☐	☐	☐	initial Mineral N
Soil texture	☐	☐	☐	☐	☐	☐	☐	☐	☐	☐	☐	☐	☐	☐	☐	☐	☐	FC, WFPS, CEC
Soil texture	☐	☐	☐	☐	☐	☐	☐	☐	☐	☐	☐	☐	☐	☐	☐	☐	☐	Other soil information
Bulk density	☐	☐	☐	☐	☐	☐	☐	☐	☐	☐	☐	☐	☐	☐	☐	☐	☐	initial SOC stock
Bulk density	☐	☐	☐	☐	☐	☐	☐	☐	☐	☐	☐	☐	☐	☐	☐	☐	☐	initial SON stock
Bulk density	☐	☐	☐	☐	☐	☐	☐	☐	☐	☐	☐	☐	☐	☐	☐	☐	☐	pH
Bulk density	☐	☐	☐	☐	☐	☐	☐	☐	☐	☐	☐	☐	☐	☐	☐	☐	☐	initial Mineral N
Bulk density	☐	☐	☐	☐	☐	☐	☐	☐	☐	☐	☐	☐	☐	☐	☐	☐	☐	FC, WFPS, CEC
Bulk density	☐	☐	☐	☐	☐	☐	☐	☐	☐	☐	☐	☐	☐	☐	☐	☐	☐	Other soil information
SOC stock	☐	☐	☐	☐	☐	☐	☐	☐	☐	☐	☐	☐	☐	☐	☐	☐	☐	initial SON stock
SOC stock	☐	☐	☐	☐	☐	☐	☐	☐	☐	☐	☐	☐	☐	☐	☐	☐	☐	pH
SOC stock	☐	☐	☐	☐	☐	☐	☐	☐	☐	☐	☐	☐	☐	☐	☐	☐	☐	initial Mineral N
SOC stock	☐	☐	☐	☐	☐	☐	☐	☐	☐	☐	☐	☐	☐	☐	☐	☐	☐	FC, WFPS, CEC
SOC stock	☐	☐	☐	☐	☐	☐	☐	☐	☐	☐	☐	☐	☐	☐	☐	☐	☐	Other soil information
SON stock	☐	☐	☐	☐	☐	☐	☐	☐	☐	☐	☐	☐	☐	☐	☐	☐	☐	pH
SON stock	☐	☐	☐	☐	☐	☐	☐	☐	☐	☐	☐	☐	☐	☐	☐	☐	☐	initial Mineral N
SON stock	☐	☐	☐	☐	☐	☐	☐	☐	☐	☐	☐	☐	☐	☐	☐	☐	☐	FC, WFPS, CEC
SON stock	☐	☐	☐	☐	☐	☐	☐	☐	☐	☐	☐	☐	☐	☐	☐	☐	☐	Other soil information
pH	☐	☐	☐	☐	☐	☐	☐	☐	☐	☐	☐	☐	☐	☐	☐	☐	☐	initial Mineral N
pH	☐	☐	☐	☐	☐	☐	☐	☐	☐	☐	☐	☐	☐	☐	☐	☐	☐	FC, WFPS, CEC
pH	☐	☐	☐	☐	☐	☐	☐	☐	☐	☐	☐	☐	☐	☐	☐	☐	☐	Other soil information
Mineral N	☐	☐	☐	☐	☐	☐	☐	☐	☐	☐	☐	☐	☐	☐	☐	☐	☐	FC, WFPS, CEC
Mineral N	☐	☐	☐	☐	☐	☐	☐	☐	☐	☐	☐	☐	☐	☐	☐	☐	☐	Other soil information
FC, WFPS, CEC	☐	☐	☐	☐	☐	☐	☐	☐	☐	☐	☐	☐	☐	☐	☐	☐	☐	Other soil information

Consistency	Progress	Error
#N/A	0.00%	

Overall Progress

0.00%

Using the judgement scale below, please fill in the pairwise questionnaire of the categories judging the <u>degree of influence</u> that each category may have on the other categories.
Each line of the pairwise should be answered twice in relation to the categories listed in column A and B. Please use the diagram on the right to explore the input included in each category. The progress column on the right of the table highlights the correct completion of the questionnaire. A yellow flag highlights incorrect completion, a green flag with "OK" highlights correct completion.
To answer the first line of the questionnaire, select on the left side of the table the level of influence (from 0 to 4) that the category "General site information" may have on the category "Climate", and using the right side of the table select the level of influence (from 0 to 4) that the category "Climate" may have on the category "General site information". If both categories have no influence on each other select the middle value 0.

PLEASE FILL IN THE QUESTIONNARIE BELOW REFERRING TO THE ECOSYSTEMS AND GHGs YOU SUBMITTED IN THE INTER-COMPARISON

Judgement scale to use in the pariwise comparisons of the broad categories		
Degree of Influence	*Definition*	*Explanation*
0	No	When a category has no influence on another one
1	Low	When a category has low influence on another one
2	Moderate	When a category has moderate influence on another one
3	High	When a category has high influence on another one
4	Very High	When a category has very high influence on another one

PAIRWISE QUESTIONARRIE OF CATEGORIES

CATEGORY A	More Influence				No Influence	More Influence				CATEGORY B	Progress
	4	3	2	1	0	1	2	3	4		
General site Information	☐	☐	☐	☐	☐	☐	☐	☐	☐	Climate	
General site Information	☐	☐	☐	☐	☐	☐	☐	☐	☐	Long-term Climate	
General site Information	☐	☐	☐	☐	☐	☐	☐	☐	☐	Experimental Data From Site	
General site Information	☐	☐	☐	☐	☐	☐	☐	☐	☐	Management Practices During Experiment	
General site Information	☐	☐	☐	☐	☐	☐	☐	☐	☐	Long-Term Management Practices	
General site Information	☐	☐	☐	☐	☐	☐	☐	☐	☐	Soil Information	
Climate	☐	☐	☐	☐	☐	☐	☐	☐	☐	Long-term Climate	
Climate	☐	☐	☐	☐	☐	☐	☐	☐	☐	Experimental Data From Site	
Climate	☐	☐	☐	☐	☐	☐	☐	☐	☐	Management Practices During Experiment	
Climate	☐	☐	☐	☐	☐	☐	☐	☐	☐	Long-Term Management Practices	
Climate	☐	☐	☐	☐	☐	☐	☐	☐	☐	Soil Information	
Long-term Climate	☐	☐	☐	☐	☐	☐	☐	☐	☐	Experimental Data From Site	
Long-term Climate	☐	☐	☐	☐	☐	☐	☐	☐	☐	Management Practices During Experiment	
Long-term Climate	☐	☐	☐	☐	☐	☐	☐	☐	☐	Long-Term Management Practices	
Long-term Climate	☐	☐	☐	☐	☐	☐	☐	☐	☐	Soil Information	
Experimental Data From Site	☐	☐	☐	☐	☐	☐	☐	☐	☐	Management Practices During Experiment	
Experimental Data From Site	☐	☐	☐	☐	☐	☐	☐	☐	☐	Long-Term Management Practices	
Experimental Data From Site	☐	☐	☐	☐	☐	☐	☐	☐	☐	Soil Information	
Management Practices During Experiment	☐	☐	☐	☐	☐	☐	☐	☐	☐	Long-Term Management Practices	
Management Practices During Experiment	☐	☐	☐	☐	☐	☐	☐	☐	☐	Soil Information	
Long-Term Management Practices	☐	☐	☐	☐	☐	☐	☐	☐	☐	Soil Information	

S5. Interview schedule

How Modellers Model

Modellers' Behaviour Study Interview Schedule

Hello, my name is {name} and I am here on behalf of the University of Aberdeen. Let me start by thanking you for agreeing to take part in this discussion on the work you do as an environmental modeller. There are no right or wrong things to say today, every opinion is valid and welcome. This study is intended to find out a little bit more about how modellers actually do their work, and in particular how they feel about the the intercomparison study that you took part in.

I will be recording the session with a Dictaphone, so if you could speak clearly that would be appreciated. Any personal identifying data that you give us, such as your name, will be anonymised before any publication of data. We keep all of your data secure and access is limited to the research team and for auditing purposes.

Do you have any questions before we begin?

1. Can you explain to me how you approach modelling?

What is your academic background?

How did you learn how to model?

Is there a correct way to model?

Has your approach evolved over time?

How do you deal with missing data?

To what extent do you allow yourself to be guided by experimental data? Or do you 'trust' the model you use?

How important is it to you that your model matches experimental data sets?

Does your answer have any impact on the 'publishability' of your work?

Is some data more important than others?

Does the model you use have an impact on your approach?

2. Which individuals or groups have an impact on the way you model?

How much importance do you place on previously published work that uses your model?

Do you work in the same way as your supervisor/boss/co-workers?

Have you ever noticed changes in your approach when moving institutions/jobs?

If you were to teach someone how to model, would you expect them to do it exactly how you do?

Who do you consider to be the biggest names in your field?

3. What can make your work either easier or more difficult?

Do you have coding knowledge? If so, do you use it when modelling? If not, do you think it would be a useful skill for your work?

Do you feel your training as a modeller has been adequate? What additional training would you like?

Do you have any experimental experience? How useful do you think this would be?

Do you think more standardised experimental data would help with modelling?

Do you have enough time to do your job?

If you had unlimited time, money and freedom, do you think your approach would change?

4. Can you tell me your thoughts on the intercomparison study?

5. Did your approach to the intercomparison differ from your usual approach?

What did you think of the anonymity of results?

Did you learn anything about your approach from this study?

Would you like to take part in a similar study in the future?

What would you change about the study?

Ignoring your results, what did you think was the most important stage of the study?

Is there anything you think I should have asked you today, that I haven't?

6. Clarifying questions for Fabrizio's pairwise study

S6. Multi-Criteria Decision Making methodology

In the first step of MCDM, we applied DEMATEL to assess the central role of each data set categories and the influence to each other. In the pairwise comparison matrix of DEMATEL, the degree of influence (d_{ij}) between category *i* and category *j* was assessed using a five point ratio judgement scale. The judgement scales 0, 1, 2, 3 and 4 represent the range from "no influence", "low influence", "medium influence", "high influence" to "very high influence", respectively. The judgement scores, received from each modeller, for the data set categories and input variables were then organized into a direct-relationship matrix ($DRM = d_{ij}$) which was normalized in *N* using Equations (1) and (2):

$$N = S \times \text{DRM} \tag{1}$$

$$S = min\left[\frac{1}{max\sum_{j=1}^{7} d_{ij}}, \frac{1}{max\sum_{i=1}^{7} d_{ij}}\right] i, j \in \{1, 2, \ldots, 7\} \tag{2}$$

where all elements in *N* comply with $0 \leq d_{ij} \geq 1$, $0 \leq \sum_{j=1}^{7} d_{ij} \geq 1$, and at least one category i such that $\sum_{j=1}^{7} d_{ij} \leq S$. The normalized matrix *N* was then used to calculate the total-relation matrix (*T*) using Equation (3):

$$T = N + N^2 + N^3 + \ldots + N^h = N(I - N)^{-1}, \quad \text{when } h \to \infty \tag{3}$$

where $T=[t_{ij}]$, for $i,j=1,2,\ldots,7$, and *I* denotes an identity matrix of *T*. In the matrix *T* the sum of the total relation vectors (t_{ij}) across the rows corresponded to the level of direct and indirect effect (G) that each input category *i* has on other categories. The sum of the total relation vectors across the columns (R) corresponded to the direct and indirect effect that category *j* receives from other categories. The sum (G+R) represents the central role that each category plays across the system and corresponds to the degree of "dominance" of the input category. While, the difference (G-R) represents the "relationship" between categories and estimates the net effect that each category contributes across the system. Data set categories with a positive G-R have a net influence on other categories and are denoted as "influential" categories. Similarly, data set categories with negative G-R are influenced by other categories and are denoted as "receiver" categories. The values of dominance and relationship between each categories were graphically reported in the influential relation diagram to provide insights on the decision making of the modellers during the multi-stage model comparison.

In the second step of MCDM, we applied ANP to assess the dependencies between input variables in the same category (inner dependencies) and the relationship between input variables in different categories (outer dependencies).

ANP, in particular, structures the modelling system as a network of dependencies and feedbacks between different data set categories. Using the linear importance ratio-scale reported in Table S1, the

modellers conducted a number of pairwise questionnaires to quantify the importance of each input category against each other, as well as the importance of the input variables within each category.

Judgement scale	Definition	Explanation
1	Equal importance	Two factors contribute equally to the objective
3	Moderate important	Experience and judgement slightly favour one factor over another
5	Strong importance	Experience and judgement strongly favour one factor over another
7	Very strong importance	Experience and judgement very strongly favour one factor over another
9	Extreme importance	The evidence of favouring one factor over another is of the highest possible order of affirmation
2,4,6,8	Intermediate values	When compromise is needed

Table S.1: Nine point linear judgement scale used in the pairwise comparison matrices to represent weight ratios among different model input variables used in the multi-stage intercomparison protocol.

The judgement scores were organized into reciprocal pairwise comparison $n \times n$ matrices for the data set categories (PCM_{cat}) and specific input variables (PCM_{var}), and resulted positive if the comparison scores (a_{ij}) are $a_{ii} = 1$, $a_{ij} > 0$, and $a_{ij} = {}^{1}/{a_{ji}}$ for all positive integer i and j (Eq. 4).

$$PCM = \begin{bmatrix} 1 & a_{12} & a_{13} & \cdots & a_{1n} \\ {}^{1}/{a_{12}} & 1 & a_{23} & \cdots & a_{2n} \\ {}^{1}/{a_{13}} & {}^{1}/{a_{23}} & 1 & \cdots & \cdots \\ \cdots & \cdots & \cdots & \cdots & \cdots \\ {}^{1}/{a_{1n}} & {}^{1}/{a_{2n}} & \cdots & \cdots & 1 \end{bmatrix} = \begin{bmatrix} {}^{w_1}/{w_1} & {}^{w_1}/{w_2} & {}^{w_1}/{w_3} & \cdots & {}^{w_1}/{w_n} \\ {}^{w_2}/{w_1} & {}^{w_2}/{w_2} & {}^{w_2}/{w_3} & \cdots & {}^{w_2}/{w_n} \\ {}^{w_3}/{w_1} & {}^{w_3}/{w_2} & {}^{w_3}/{w_3} & \cdots & {}^{w_3}/{w_n} \\ \cdots & \cdots & \cdots & \cdots & \cdots \\ {}^{w_n}/{w_1} & {}^{w_n}/{w_2} & \cdots & \cdots & {}^{w_n}/{w_n} \end{bmatrix} = n \begin{bmatrix} w_1 \\ w_2 \\ w_3 \\ \cdots \\ w_n \end{bmatrix} \quad (4)$$

When a modeller is perfectly consistent in his/her judgements in the pairwise comparison, the importance vectors (w) between two elements is perfectly consistent as $a_{ij} = \frac{w_i}{w_j}$ and $w_i = w_j$. Due to small inconsistencies in the judgements, a_{ij} can only be estimated, leading to eigenvalue problems in A in the form $Aw = \lambda_{max} w$, where λ_{max} is the principal eigenvalue. Cardinal inconsistency in the *PCMs*, therefore, was measured as $\lambda_{max} - n$, which measures the deviation of the judgment from the consistent approximation (Saaty, 1990). Prior to acceptance of the results, the *PCMs* were tested for consistency using the consistency index (*CI*) and consistency ratio (*CR*) (Eq. 5, 6):

$$CI = \frac{\lambda_{max} - n}{n-1}, \text{ if the matrix is perfectly consistent } CI = 0. \quad (5)$$

$$CR = \frac{CI}{RI} \quad (6)$$

where λmax the largest eigenvalue in the normalized *PCMs* of each input category (Eq. 4), and *RI* is the average value of *CI* calculated from randomly generated *PCMs* (Alonso and Lamata 2006). *PCMs*

with CR ≤ 0.15 were considered acceptable (i.e. 15% consistency limit), and *PCMs* with CR > 0.10 were reviewed by returning the *PCMs* to the modellers prior a telephone interview to discuss their results.

In PCM_{cat} the judgement scores were normalized for each category so that each column sums to 1 (w_{ij}), and multiplied by the total relation vectors (t_{ij}) from step 1 to form a stochastic weighted supermatrix (WS) (Eq. 7):

$$WS = \begin{array}{c} C_1 \\ \vdots \\ C_i \\ \vdots \\ C_n \end{array} \overset{\begin{array}{ccccc} C_1 & \cdots & C_j & \cdots & C_n \end{array}}{\begin{bmatrix} w_{11} \times t_{11} & \cdots & w_{1j} \times t_{1j} & \cdots & w_{1n} \times t_{1n} \\ \vdots & & \vdots & & \vdots \\ w_{i1} \times t_{i1} & \cdots & w_{ij} \times t_{1j} & \cdots & w_{in} \times t_{in} \\ \vdots & & \vdots & & \vdots \\ w_{n1} \times t_{n1} & \cdots & w_{nj} \times t_{nj} & \cdots & w_{nn} \times t_{nn} \end{bmatrix}} \tag{7}$$

where C_n is the nth input category, w_{ij} is the principal weighted eigenvector of the importance of jth category compared to the ith category, and t_{ij} is the total relation vector between the jth category compared to the ith category. To calculate the final importance weights of each data set categories (W_i), *WS* was raised to its limiting powers (k) until the relative weights converged to stable values forming the limiting supermatrix (LS) (Eq.8).

$$LS = \lim_{k \to \infty} PCM_{WS}{}^{k} \tag{8}$$

In PCM_{var} the importance weights of the input variables were calculated using the additive normalization method (Saaty 1980). The pair-wise comparison scores (a_{ij}) of the input variables were divided by the sum of all elements in the columns, summed across the rows to calculated the normalized eigenvalues, and divided by the number of elements n in the matrices to obtain the importance weights of each input variable (p_i) (Eq. 9):

$$\bar{a}_{ij} = \frac{a_{ij}}{\sum_i a_{ij}} \tag{8}$$

$$p_i = \frac{1}{n}\sum_j \overline{a}_{ij} \tag{9}$$

Successively, the importance weights p_i were multiplied by the categorical weights W_i to calculate the final ANP weighted priorities of the input variables (P_i) across the whole modelling framework (Eq. 10).

$$
\begin{array}{cc|ccccc|}
 & & C_1 & \cdots & C_j & \cdots & C_n \\
 & & v_{11} \cdots v_{1m_1} & & v_{j1} \cdots v_{jm_j} & & v_{n1} \cdots v_{nm_n} \\
 & v_{11} & & & & & \\
C_1 & \vdots & & & & & \\
 & v_{1m_1} & W_{11} \times p_{11} & \cdots & W_{1j} \times p_{1j} & \cdots & W_{1n} \times p_{1n} \\
\vdots & & \vdots & & \vdots & & \vdots \\
 & v_{i1} & W_{i1} \times p_{i1} & \cdots & W_{ij} \times p_{1j} & \cdots & W_{in} \times p_{in} \\
C_i & \vdots & \vdots & & \vdots & & \vdots \\
 & v_{im_1} & & & & & \\
\vdots & & W_{n1} \times p_{n1} & \cdots & W_{nj} \times p_{nj} & \cdots & W_{nn} \times p_{nn} \\
 & v_{n1} & & & & & \\
C_n & \vdots & & & & & \\
 & v_{nm_n} & & & & &
\end{array} \tag{10}
$$

where C_n is the nth input category, v_{nm} is the mth input variable in the nth category.

S7. Table S2

Summary of the opinion of the modellers that used the APSIM, DairyMod, and DayCent about the importance of the input variables, and the level of influence given and received (i.e. relationship) by each data set category in the modelling protocol. Categories with positive relationship have a net influence towards the value of other data set categories, and are denoted as influential categories. The values in brackets outline the level of Kendal concordance between the modellers, and * indicates significant concordance at the $p < 0.05$ level. N corresponds to the number of modellers that used each model type.

Input Category (APSIM - DairyMod - DayCent)	Input variable	Importance			Relationship		
		APSIM	DairyMod	DayCent	APSIM	DairyMod	DayCent
		n = 2	n = 4	n = 4			
**General site information** (0.88* - 0.18 - 0.76*)	Crop type (crop rotation)	0.48	0.2	0.4	0.31	0.02	-0.16
	Mean regional yield	0.15	0.26	0.29			
	Experimental length and season	0.27	0.24	0.17			
	Location (country, latitude N)	0.05	0.18	0.09			
	Terrain info	0.05	0.11	0.05			
**Climate during experiment** (0.84* - 0.25 - 0.78*)	Precipitation	0.28	0.25	0.36	1.53	0.97	0.61
	Air temperature	0.26	0.23	0.25			
	Solar radiation	0.32	0.2	0.21			
	Other climate factors (Wind, [CO_2])	0.06	0.14	0.07			
	Air humidity	0.04	0.12	0.06			
	Atm. pressure	0.04	0.06	0.04			
**Long-term Climate** (0.46 - 0.29 - 0.96*)	Air temperature	0.22	0.22	0.27	1.16	0.99	0.62
	Precipitation	0.22	0.22	0.34			
	Solar radiation	0.22	0.18	0.21			
	Air humidity	0.11	0.1	0.07			
	Atm. pressure	0.11	0.1	0.04			
	Other climate factors (Wind, [CO_2])	0.11	0.18	0.08			
**Experimental data from site** (0.13 - 0.11 - 0.10)	Annual extracted yield	0.28	0.26	0.12	-1.1	-0.79	-1.45
	Dynamic soil moisture	0.1	0.11	0.13			
	Vegetation data (Phenology, LAI)	0.12	0.1	0.03			
	N_2O and/or CH_4	0.09	0.08	0.15			
	GPP & NEP	0.05	0.1	0.14			
	NEE & Reco	0.04	0.11	0.13			
	Dynamic soil mineral N	0.13	0.04	0.07			
	Dynamic SOC & SON stock	0.04	0.06	0.12			
	Soil N losses	0.05	0.07	0.07			
	Dynamic soil temperature	0.09	0.04	0.04			
**Management practices during experiment** (0.24 - 0.04 - 0.16)	Fertilization rates	0.26	0.14	0.26	-1.0	-0.31	-0.73
	Irrigation	0.22	0.12	0.19			
	Freq. harvest, grazing & cut in grass	0.13	0.15	0.12			
	Fertilizer type	0.07	0.09	0.1			
	Frequency of ploughing	0.06	0.1	0.09			
	Crop residues	0.08	0.09	0.08			
	Intercropping	0.09	0.1	0.06			
	Fertilization mode	0.07	0.09	0.05			
	Frequency of other activities	0.02	0.11	0.04			
**Long-term management practices** (0.13 - 0.02 - 0.12)	Fertilization rates	0.2	0.11	0.21	-0.55	-0.64	-0.53
	Irrigation	0.17	0.11	0.16			
	Frequency of harvest	0.11	0.09	0.11			
	Intercropping	0.13	0.09	0.06			
	Land use history	0.05	0.1	0.09			
	Frequency of ploughing	0.05	0.1	0.09			
	Crop residues	0.12	0.1	0.08			
	Fertilizer type	0.05	0.07	0.09			
	Frequency of other activities	0.07	0.11	0.03			
	Fertilization mode	0.03	0.08	0.04			
**Soil information** (0.54 - 0.12 - 0.45)	Soil texture	0.09	0.12	0.26	-0.37	-0.24	-0.89
	FC, WFPS, CEC	0.27	0.2	0.2			
	Bulk density	0.09	0.15	0.14			
	Initial SOC stock	0.15	0.11	0.14			
	pH	0.14	0.09	0.09			
	Initial SON stock	0.15	0.11	0.04			
	Soil mineral N	0.06	0.08	0.05			
	Other soil information	0.02	0.09	0.05			
	Soil type	0.02	0.06	0.03			

Footnote: The colour gradient indicates where the relative importance of each input variables falls within each data set category.
